# Counterion-Mediated Humidity Modulation of Electronic Transport in Pristine MWCNT/TOCNF Composites

**DOI:** 10.1021/acsomega.6c04065

**Published:** 2026-10-02

**Authors:** Andreza J. Siqueira, Wflander M. Souza, Rayssa O. Matos, Hana H. Koga, Vinicius Ornelas, Claudio Laudares, Dalila C. Sicupira, Thiago Colla, Ive Silvestre, Ana Paula M. Barboza

**Affiliations:** † Department of Physics, 28115 Universidade Federal de Ouro Preto, Ouro Preto 35400-000, MG, Brazil; ‡ Department of Chemistry, Universidade Federal de Ouro Preto, Ouro Preto 35400-000, MG, Brazil; § CTNano, Universidade Federal de Minas Gerais, Belo Horizonte 31270-901, MG, Brazil; ∥ Department of Physics, Universidade Federal de Minas Gerais, Belo Horizonte 31270-901, MG, Brazil

## Abstract

Humidity-driven electrical responses in carbon-cellulose composites are commonly attributed to matrix swelling. However, in TEMPO-oxidized nanocellulose (TOCNF), water uptake also releases Na^+^ counterions from carboxylate residues, enabling interfacial polarization via image-charge interactions that can modulate junction-limited electronic transport. Here, we formulate aqueous inks in which TOCNF disperses pristine (nonfunctionalized) multiwalled carbon nanotubes (MWCNT) without covalent modification, yielding homogeneous, spray-processable composite films. Using DC electrical measurements under controlled relative humidity, impedance spectroscopy, scanning probe microscopy, and a mean-field electrostatic model for counterion-mediated polarization, we show that percolated TOCNF/MWCNT films exhibit reproducible resistance gains, reaching up to ∼270%, that are inconsistent with a swelling mechanism alone. To support this view, a control based on nonoxidized nanocellulose (with a negligible amount of dissociable counterions) was designed, which shows only modest changes at high humidity, thereby sustaining an important, counterion-mediated interfacial route. The impedance data indicate predominantly electronic transport and a humidity-dependent low-frequency reactive response near the percolation threshold. The DC measurements establish a reproducible steady-state behavior and within-session cycling, while intrinsic response times and long-term stability remain to be more explored. The model rationalizes how increased counterion concentration under humidification can induce polarizing charges at the nanotube surface, driving electrostatic repulsion and relative reorientation that weaken conductive CNT-CNT junctions across the network. Invoking the Derjaguin approximation, an effective, counterion-induced repulsive interaction between CNT surfaces is computed, which prevents the building-up of large connecting junctions across the network, thus reducing their ability to assemble percolating pathways. These findings establish an overlooked counterion-mediated design principle to tune mixed ionic-electronic transport in sustainable CNT/nanocellulose electronic composites operating under humid or electrolyte-adjacent conditions.

## Graphical abstract

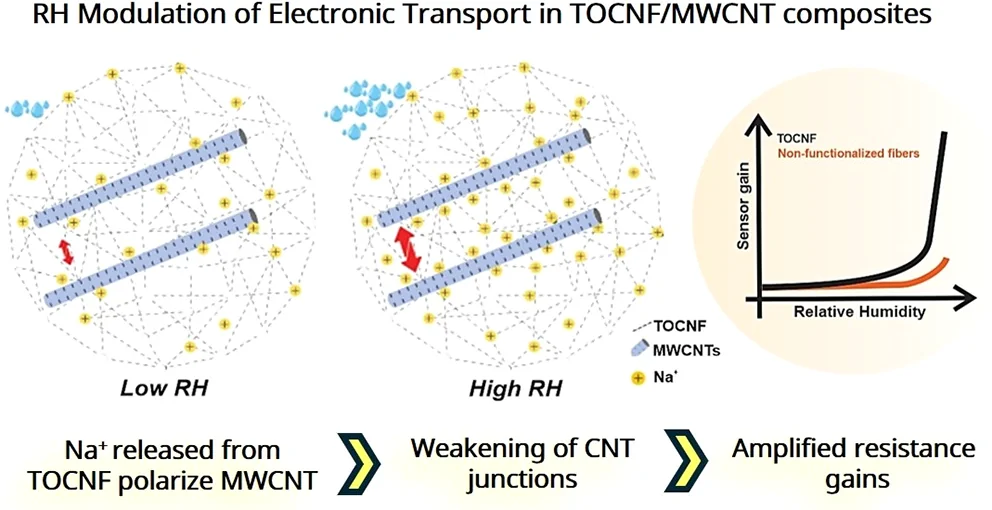

## 1 Introduction

Cellulose-derived substrates (nanopaper and cellulose papers) have enabled a class of flexible, paper-based platforms for humidity-responsive devices.^1–5^ When combined with nanostructured conductive coatings, these substrates provide mechanically compliant architectures that remain compatible with low-cost processing. Multiwalled carbon nanotubes (MWCNTs), used in immobilized configurations, are particularly effective conductive fillers because their high aspect ratio supports percolated pathways with robust electronic transport even at very small packing fractions, which is essential for wearable formats and packaging-oriented implementations.^4,6–11^

Cellulose, a sustainable, affordable, and biodegradable material, has attracted significant research interest for applications in nanotechnology.^12–15^ As a natural polymer derived from plants and bacteria, cellulose exhibits mechanical robustness and rich interfacial chemistry, enabling materials relevant to optoelectronics, biosensors, and energy storage applications.^16–21^ The water affinity characteristic of most cellulose-based materials is central to their use in humidity-responsive systems: water uptake from the surrounding vapor induces conformational changes and swelling of the polymer matrix.^22–24^ Direct readout of such mechanical stimuli would require techniques able to resolve small strain variations at a nanoscale level, which becomes unfeasible in most practical, large-scale implementations.^25–27^ An alternative is to rely on indirect electrical responses to water adsorption – such as changes in current or charge-transport fluctuations – which are accessible with standard instrumentation. Although cellulose matrices can sustain ionic conduction, their high impedance hampers practical readout. Incorporating conductive fillers is therefore important in providing an electronically percolating pathway that lowers the baseline resistance to measurable levels. In this context, carbon nanotubes (CNTs) are natural candidates owing to their excellent electrical and thermal conductivities, mechanical robustness, high aspect ratio, and compatibility with numerous natural (as well as synthetic) polymers, enabling nanocomposites with controllable responses to external stimuli.^28,29^

Humidity-driven electrical responses in cellulose–carbon nanotube composites are commonly attributed to polymer swelling and geometrical rearrangement of the conductive network as water is adsorbed.^30^ In polyelectrolyte nanocellulose matrices, however, water uptake also promotes ionic dissociation and mobilizes counterions, notably Na^+^ in TEMPO-oxidized nanocellulose.^31^ Although this ionic response is established for TOCNF itself, how mobile counterions affect electronic transport through CNT–CNT junctions in a percolated composite remains unresolved.

Cellulose/CNT humidity sensors already encompass several material and device strategies. Zhu et al. used TEMPO-oxidized cellulose nanofibers both to disperse CNTs in water and as the humidity-responsive phase in vacuum-filtered films, reporting a maximum current response of 69.9% at 95% RH.^9^ Related studies have used cellulose/CNT fibers, porous or bilayer networks, electrostatic assembly, and patterned deposition to increase water access or improve network durability.^8,11,32^ The closest chemical analogue is the TOCNF/oxidized-CNT film of Solin et al., whose resistance increased by approximately 15% between 20 and 60% RH and was interpreted mainly through water uptake and swelling of the fibrillar network.^33^

These reports establish that neither an increase in resistance with humidity nor the use of charged nanocellulose to disperse CNTs is, by itself, new. Related CNT/cellulose composite fibers and sulfonated-CNT/nanocellulose hydrogels further show how nanotube surface chemistry and matrix organization affect interfacial compatibility and network connectivity.^34–36^ More broadly, humidity transduction has also been achieved through ionic conduction in cross-linked poly­(ionic liquid)­s,^37^ proton hopping coupled to a ZnO/graphene heterojunction,^38^ and swelling-induced strain in carbon-nanofiber/MWCNT-coated fiber Bragg gratings.^39^ These examples show why response magnitude or sign alone cannot identify the underlying mechanism: hydration may change the electronic properties of the carbon phase, rearrange a percolating network, activate ionic transport through the matrix, or be converted into strain by the device architecture.

Bridging this gap requires controlling both the ionic environment of the nanocellulose matrix and the connectivity of the CNT network in water-processable formulations. In practice, however, translating CNT-based composites into water-processable formulations remains challenging, mainly due to the hydrophobic nature of CNTs, which leads to irreversible aggregation that prevents the use of most of these composites in solution–an essential requirement for producing inks, sprays, foams, and aerosols.^40^ Combined with short-range van der Waals attractions, hydrophobic effects lead to CNT aggregation when dissolved in polar solvents, strongly limiting their applicability in water-based systems.^41^ One standard way to overcome this drawback relies on chemical functionalization methods – such as acid treatments introducing carboxyl and hydroxyl groups – which have been successfully employed to enhance CNT dispersibility in water. However, these methods can alter the intrinsic properties of CNTs and involve environmentally hazardous reagents. In addition, the absence of proper dispersion steps can significantly impair the performance and uniformity of CNT-based composites.^41^ As an environmentally friendly and lower-cost alternative, nanocellulose – especially TOCNF – has been studied as a dispersant agent for carbon materials, including MWCNTs. TOCNF can promote CNT stabilization through a combination of electrostatic and steric repulsions, enabling dispersion even in the absence of covalent functionalization.^25,42–45^ Combined with ultrasonic baths, studies have demonstrated the possibility to produce homogeneous and stable mixtures of these materials.^9,25,42,43,46,47^ Cellulose/CNT composites can thus be fabricated into various forms, such as thin films, fibers, foams, and aerogels, making them suitable for many applications such as in sensor devices, batteries, and supercapacitors.^46–50^ Despite these clear advantages, the mechanisms connecting such dispersive forces to charge transport in nanocomposites of varying water content are not yet clear. To rationalize how these aqueous formulations translate into electrical performance, we herein consider a microscopic perspective that links interfacial physics to network connectivity.

From a theoretical perspective, the description of charge transport across structures composed of a set of conducting particles embedded in an amorphous, nonconducting polymeric matrix, poses many challenging questions.^51–56^ Of fundamental relevance in this context is the question of whether or not charge transport between source and drain is able to be established. This is the key question behind the classical percolation theory, which establishes the conditions under which an event corresponding to a fully connected network of conducting channels spanning the whole system is likely to take place.^56–61^ Neglecting mobility effects of (randomly sized) CNTs embedded into an isolating matrix, a percolating transition is established at the so-called *percolation threshold*, that depends only on the packing fraction of insertions (conducting elements) making up the composite. Due to their high aspect ratios, CNTs have low percolation thresholds (typically around 1%), a property that justifies their growing success as basic building blocks in nanocircuit applications.^62–64^ In practice, this percolative picture resolves at the scale of intertube junctions, where local barriers and random contact statistics control macroscopic transport. When particle interactions become non-negligible, deviations from such a scenario of fully random insertions should give rise to nontrivial transport phenomena, such that deviations from classical percolating theory predictions are likely to occur.^57,65–68^

When absorbed into a nonconducting polymeric matrix, a small amount of CNTs is sufficient to fully promote charge transport between connecting electrodes. In this case, the percolating network can be envisioned as a set of (randomly arranged) conducting lines, some of which are linked by “junctions”, i.e., intersection points where neighboring CNTs are close enough to allow electron tunneling through the embedded matrix. Such a “hopping” mechanism requires large energy fluctuations to surmount an energetic barrier which depends on the closest distance between CNTs, their size and structure, as well as the conducting properties of the polymeric matrix.^69^ For this reason, they can be described as resistance sites that allow for charge transport through individual segments of the connecting network. Even at concentrations far beyond the percolation threshold, it is reasonable to expect the conducting response of the composite to be strongly dependent on the overall amount of such junctions, as well as on the typical energy barrier between those gaps. These properties clearly depend on the spatial distribution of conducting particles on the composite: CNTs lying closer together with a larger contact area will more likely exchange their charge carriers under thermal fluctuations, thus enhancing the overall connectedness of the network.^70–72^ Theoretical models aimed to describe such complex distributions mostly rely on the idea of random “inclusions” of conducting CNTs onto a background matrix of poor conductors. This allows for fairly accurate estimations of macroscopic properties based on simple inputs such as relative concentrations, particle sizes, shapes, and polydispersity.^73–75^ Macroscopic properties such as effective conductivity or equivalent resistances are then extracted under suitable volume averages, or via the application of Kirchhoff’s laws.^57,69,76,77^ Most percolation approaches rely solely on exclusion volume interactions between inclusions and the embedding matrix. However, geometry alone cannot capture interfacial forces that become relevant under humidity, prompting us to examine nonsteric interactions in these composites. Indeed, the role of interactions between inclusions beyond exclusion volume is mostly overlooked in building up the percolating networks. In many situations, however, the composite is most easily synthesized upon dilution in an aqueous solvent, followed by its thermal annealing into a substrate. A reasonable assumption in this case is that the composite will still retain some of its structural features from its solution state,^78^ mostly dominated by combined interparticle and thermal forces. Even if one assumes completely random inclusions in the initial dry state, particle interactions should play a paramount role in controlling the dynamic response in humidity devices. Increasing water adsorption triggers thermal motions,^27^ which in turn act to restore the original situation of a stable equilibrium in solution.^79,80^ In particular, the presence of mobile counterions in an increasingly wet environment might lead to structural arrangements resembling that of a charge-stabilized suspension.^81^ In such a case, electrostatic forces should avoid CNT aggregation,^42,43^ resulting in a less connected network. Taken together, humidity-induced counterion release and the resulting electrostatic forces provide a concrete pathway to reduced network connectedness beyond standard exclusion-volume effects, especially in the presence of charged nanocellulose matrices such as TOCNF.

Guided by this framework, we prepared aqueous composite inks in which TOCNF disperses as-received MWCNTs and evaluated the resulting films under controlled humidity. The formulation avoids covalent oxidation of the nanotubes and added surfactants: TOCNF provides the water-processable matrix, hygroscopic interface, and dissociable counterions, whereas the MWCNTs provide the electronically conducting network. We varied the composition across the percolation region to determine how hydration couples to the dominant transport pathway. Below the threshold, hydration lowers the resistance through matrix-assisted conduction, whereas above it the resistance increases as CNT–CNT junctions are weakened. The low-frequency impedance response near the threshold and the equilibrium polarization model provide complementary evidence that mobile charge participates in this junction-limited response. This interpretation does not exclude matrix swelling or establish the microscopic location of the ions; rather, it identifies a route by which the ionic environment of charged nanocellulose can modulate electronic connectivity in a network of unmodified MWCNTs.

To account for the observed conductivity trends within this charged matrix, we developed a simple theoretical model based on polarization by released counterions. The model qualitatively explains the observed conductivity response via the attraction of mobile counterions toward their induced image-charges at the CNTs surfaces. The building-up of such polarization-induced double-layers thus prevents the formation of charge-transferring connections between CNTs — naturally resulting from intrinsic van der Waals (vdW) adhesion — thereby reducing the available pathways that promote electron conduction across the percolating network. We examine this picture using controlled-humidity DC measurements, impedance spectroscopy, and complementary SPM (AFM/LFM/EFM) analyses. Because the model is static and adiabatic, it is used to interpret the interfacial mechanism rather than to fit the frequency-dependent impedance spectra.

## 2 Experimental and Theoretical Methods

### 2.1 Materials

Multiwalled carbon nanotubes (MWCNT) with outer diameters of *D* = 10–30 nm, lengths of *L* = 10–30 μm, and purities greater than 95% were supplied by CTNano/UFMG^82^ as received (no surfactant or functionalization). TEMPO-oxidized cellulose nanofibrils (TOCNF) were obtained from the University of Maine (USA) as a 1.0 wt % aqueous gel (Na^+^ counterion). According to the supplier’s datasheet,^83^ the carboxylate content is 0.2–2.0 mmol g^–1^ (dry basis). Gel aliquots were weighed and dry mass was calculated assuming 1.0 wt % solids; no batch-specific charge titration was performed. Additional physicochemical details and characterization data for these materials are provided in the references.

### 2.2 Scanning Probe Microscopy (SPM) Measurements

For the characterization by Scanning Probe Microscopy, a Park XE7 SPM system (Park Systems) was employed. Topographical imaging and the friction analyses were performed using Atomic Force Microscopy (AFM) and Lateral Force Microscopy (LFM), respectively. The measurements were carried out with high resolution silicon AFM cantilevers CSG10 series (TipsNano), featuring a natural resonance frequency of 22 kHz, an elastic constant (k) of 0.11 N/m, and a tip radius of curvature of approximately 10 nm. Electrical force microscopy (EFM) measurements (see SI material) were performed using a MultiMode 8, scanning probe microscope, equipped with a Nanoscope V controller (Bruker). Pt-coated silicon cantilevers (XSC11/Pt, MikroMasch), with a nominal resonance frequency of 80 kHz, a spring constant of 2.7 N/m, and a tip radius of approximately 30 nm, were used for the measurements. All experiments were conducted under ambient temperature and humidity conditions.

### 2.3 Ink Formulation

For the SPM characterizations of the individual inks, MWCNTs were dispersed in *N*-methyl-2-pyrrolidone (NMP) at 0.1 wt % (w/w), and TOCNF gel was diluted 1:100 (v/v) from the original 1.0 wt % gel using deionized (DI) water. A droplet of each solution was deposited via drop-casting onto silicon substrates consisting of 300 nm of thermally grown SiO_2_ on doped Si. After 60 s, the samples were dried with a gentle stream of dry nitrogen.

For the electrical characterization of the individual inks, CNT-only devices used the same NMP-based dispersion 0.1 wt % (w/w). TOCNF-only devices were prepared from an aqueous dispersion (0.14 wt % -w/w, dry basis). All MWCNT/TOCNF composites were water-based.

The three main composite inks investigated in this work – S1, S2, and S3 – were prepared as follows: 40 mg of nonfunctionalized MWCNT were mixed with varying amounts of aqueous TOCNF gel (1.0 wt % solids), resulting in dry mass CNT:TOCNF ratios of 1:0.5 (S1), 1:1.7 (S2), and 1:2.2 (S3). All CNT:TOCNF ratios refer to nominal dry mass computed from the weighed gel and the 1.0 wt % solids specification. DI water was then added to each formulation so that the total water mass was kept constant. Once the components were combined, the dispersions were manually stirred and sonicated in a bath sonicator (UNIQUE USC2850, 120 W, 25 kHz) for 3 h to ensure homogenization.

For the AC impedance measurements, three MWCNT/TOCNF formulations spanning the percolation region were selected, with nominal dry-solid MWCNT volume fractions of ϕ_*v*_ = 0.18, 0.21, and 0.34, corresponding to the below-threshold, near-threshold, and well-percolated regimes, respectively.

### 2.4 Device Fabrication and Film Deposition

Films were prepared by spray-coating either individual component inks or MWCNT:TOCNF hybrid formulations using a 0.5 mm airbrush pen (OneTools). Approximately 3 mL of each dispersion was sprayed over a 1.0 × 1.0 cm^2^ rectangular area between two prepatterned copper electrodes on phenolic substrates. The substrates were maintained at 95 °C during deposition to promote rapid water evaporation. Each device was built up from ∼18 sequential spray passes, with each layer deposited only after visually complete drying of the previous one. After the final pass, the films remained at 95 °C for 1 min and were then cooled back to room temperature and placed in the gas-mixing chamber for the electrical characterization.

### 2.5 Electrical Measurements under Controlled Humidity

Electrical characterization was performed under identical device layouts using the same 18-pass spray routine at 95 °C. The measurements were performed at room temperature, in a custom gas-mixing chamber, where relative humidity (RH) was adjusted by mixing dry and water-bubbled argon (Ar) streams and monitored in real-time with a colocated DHT22 sensor. Humidity was ramped periodically, from ∼10 to 95%, keeping the total gas flow at 1000 sccm. Each target humidity was held in a plateau for approximately 10 min, after which a current–voltage (*I*–*V*) curve was acquired in a two-probe configuration with a Keithley 2400 SourceMeter connected via feedthroughs; data were analyzed within the −1.5 to +1.5 V range and resistances obtained from the linear fits.

For the steady-state resistance-gain analysis, three independently coated devices were characterized for each hybrid formulation. The triplicate sets included devices fabricated with copper and gold-protected copper contact pads; the assignment for each device is provided in the Supporting Material. The resistance gain was calculated separately for each device as *R*_G_ = 100­(*R*_RH_ – *R*_0_)/*R*_0_, where *R*_0_ is the resistance at the lowest RH measured for that device. For each RH plateau, the available device values were averaged, and the error bars represent the sample standard deviation.

Step-response measurements were carried out at a fixed bias of 0.2 V while the chamber was switched between low- and high-humidity gas-flow conditions. Chamber RH and device resistance were recorded simultaneously. Response and recovery times were defined as the time required for the resistance to change from 10 to 90% of its total variation between the initial and final plateaus; no kinetic fit was used. Three consecutive increasing/decreasing-RH cycles were also recorded on one S3 device to examine within-session reversibility. The complete calculation and inclusion criteria are provided in the Supporting Information.

Impedance spectra were acquired from 20 mHz to 100 kHz using a 20 mV rms perturbation in the same humidity-controlled chamber. Three MWCNT/TOCNF films were examined: a below-threshold film (nominal dry-solid MWCNT volume fraction ϕ_*v*_ approximately 0.18), a near-threshold film (ϕ_*v*_ approximately 0.21), and a well-percolated film (ϕ_*v*_ approximately 0.34). The nominal fractions were calculated from the dry component masses and densities are composition labels.

The impedance measurements used the same two sample contacts in a coplanar configuration. The working and sense leads were paired at one contact, and the counter and reference leads at the other; protective earth remained outside the chamber and was not connected to the sample. The near-threshold device was measured sequentially at 49.0, 81.0, 90.0 and 94.5%.

### 2.6 Theoretical Background

We begin by sketching the theoretical model proposed to describe the behavior of our CNT networks in a charged nanocellulose matrix, as a basis for interpreting the experiments that follow. Employing a semiquantitative description, emphasis is placed on the mechanisms underlying indirect ionic effects (counterion-induced) on electronic transport through the percolating network, in regimes of fully wet environments. We shall outline the basic assumptions and key equations necessary to understand these mechanisms. Detailed calculations and an in-depth theoretical analysis can be found in the Supporting Information available.

We assume that the dried composite has random particle distributions, with overall concentrations coinciding with those of the original solution. As the surrounding water vapor increasingly condenses into the substrate, ionic mobility starts to set in. As a result, the system will undergo structural reorganizations in the sense of slowly restoring its aggregation properties in solution. Sodium counterions of concentration *c*_*s*_ (which clearly increases with the amount of absorbed water) are then free to move through a background of polymeric backbones and CNTs. Since the latter has much larger contact areas, it is reasonable to suppose that ion diffusion is highly influenced by ionic interactions with the large CNTs surfaces. On the other hand, the thinner TOCNF backbones can be viewed as dragging centers that try to keep sodium ions away from CNTs surfaces, as the backbones are endowed with negatively charged residues. In a mean field formulation, we shall represent the effects of such residual charges in the neighborhood of a fixed CNT as providing a uniform neutralizing background of charge ρ_*b*_ = – *c*_*s*_ *e* (where e is the elementary charge), in which sodium unassociated ions (*i.e.*, those not bound to CNTs/fibers surfaces) are free to move. The situation is depicted in Figure 1a, in which a CNT is represented as an infinitely long rod placed at the center of a cylindrical cell.

**Figure 1:**
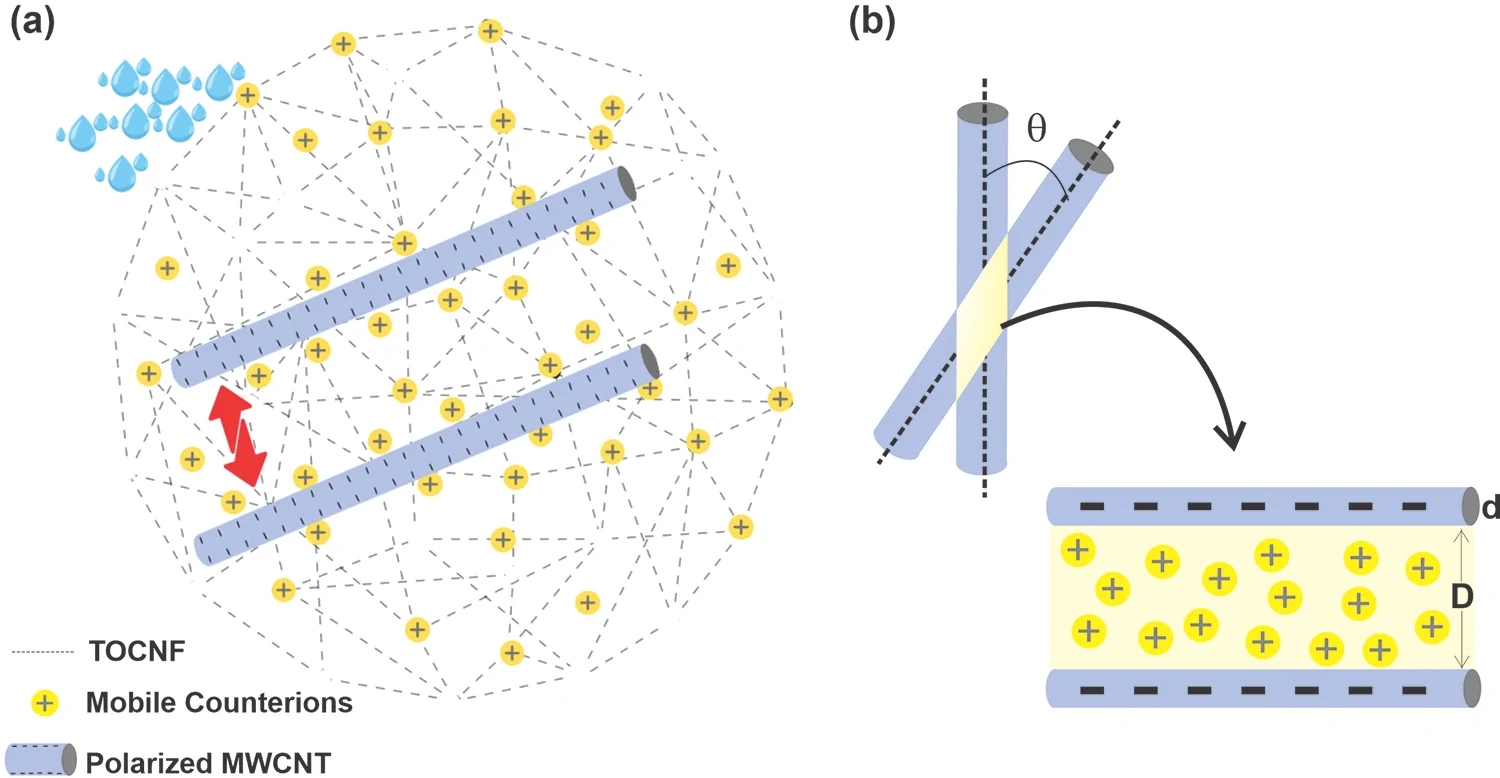
Illustrative representations of our model system. In (a), two polarized rods are separated at a typical distance which depends on their overall concentration, due to ionic-induced mutual repulsion. As a result, each nanorod can be placed at the center of a virtual cylindrical cell, representing a MWCNT and its close environment. Sodium ions (yellow spheres) of bulk concentration *c*_*s*_ are free to move across a neutralizing, rigid background of charge – *c*_*s*_ *e*, representing the overall effects of static charges on the fiber backbones. In (b), the areas facing each other of two rods of relative orientation θ are mapped into a parallel-plate system. The distance *D* between the plates corresponds to the closest surface separations, and the surface charge densities on the plates are the same as those in the rods. When *D* ≪ *d*, Derjaguin’s approximation can be employed to incorporate local curvature effects from the cylindrical surfaces.^80^

Since the inner diameters of MWCNTs are typically much smaller than the external ones, we consider, for simplicity, the MWCNTs as solid rods of radius *a* = *d*/2 (where *d* is the external diameter of the tube). As mobile ions are driven to the close vicinity of the rod surface via thermal diffusion, a strong interaction arises with its image charges induced inside the rod’s core.^84–87^ As MWCNTs usually display hybrid semiconductor/conductor behavior, the static dielectric constant *ε*_1_ in their interior is considered to be much higher than that of the aqueous environment, ε_2_ ≈ 80.

Image-charge interactions are thus attractive, leading to the formation of a condensed layer of sodium ions at the outer surface, together with an electronic depletion layer on its inner side. In practice, a second layer of “holes” should be induced at the innermost MWCNTs surface (here absent), in such a way as to keep its overall electroneutrality. Due to such polarization mechanism, the rod will behave as if it had an apparent surface charge σ_*p*_, leading to an effective double layer repulsion between approaching surfaces.

The apparent surface charge can be easily evaluated once the averaged potential ψ­(*S*) is known (where *S* is the radial distance from the rod’s center), via a simple application of Gauss law:

1 $$\sigma_{p}=-{\frac{d\psi }{dS}|}_{S=a}\frac{\varepsilon_{2}}{4\pi e}$$

Invoking a mean-field approximation, the averaged potential is related to the counterion distribution ρ­(*S*) via:

2 $$\rho \left(S\right)=c_{s}\exp[-\beta \left(e\psi \left(S\right)+W_{\mathrm{im}}\left(S\right)\right)]$$

where β = 1/*k*_B_ *T* in the inverse thermal energy, *k*_B_ the Boltzmann constant and *T* the temperature. The potential *W*_*im*_(*S*) represents the attractive surface-ion interaction, and can be evaluated using the Green function formalism as (see Supporting Information):

3 $$W_{\mathrm{im}}\left(S\right)=\frac{2e^{2}}{\pi \varepsilon_{2}}\sum_{m=0}^{\infty }\left(1-\frac{\delta_{m0}}{2}\right)\int_{0}^{\infty }dk\frac{\varepsilon_{2}qI_{m}\left(\mathrm{ka}\right)I_{m}^{\prime }\left(\mathrm{qa}\right)-\varepsilon_{1}kI_{m}\left(\mathrm{qa}\right)I_{m}^{\prime }\left(\mathrm{ka}\right)}{\varepsilon_{2}qK_{m}^{\prime }\left(\mathrm{qa}\right)I_{m}\left(\mathrm{ka}\right)-\varepsilon_{1}kK_{m}\left(\mathrm{qa}\right)I_{m}^{\prime }\left(\mathrm{ka}\right)}\frac{K_{m}^{2}\left(\mathrm{qS}\right)}{q^{2}S^{2}}$$

$q\equiv \sqrt{\kappa^{2}+k^{2}}$ , $\kappa =\sqrt{4\pi \lambda_{B}c_{s}}$ defines the inverse Debye screening length for a one-component plasma, λ_*B*_ = β*e* ^2^/ε_2_ defines the Bjerrum length, and the functions *I*_*m*_(*x*) and *K*_*m*_(*x*) are *m*–th order modified Bessel functions of first and second kinds, respectively. We note that the potential *W*_*im*_(*S*) is of short-range, as the interactions between an ion and its image-charges become rapidly screened by intervening ions. In practice, this potential also comprises repulsive interactions resulting from an increased ionic self-energy close to the interface, where screening is less effective than in bulk solution due to polarization of the ionic cloud (the so-called solvation energy). Equations 2 and 3 can be combined with the Poisson equation, leading to the following modified Poisson–Boltzmann (mPB) equation:

4 $$\nabla^{2}\mathrm{\psi ̃}=-\kappa^{2}\left[e^{-\left(\mathrm{\psi ̃}\left(S\right)+{\mathrm{W̃}}_{\mathrm{im}}\left(S\right)\right)}-1\right]$$

where ψ̃ ≡ β*eψ* and W̃_*im*_ ≡ β*W*_*im*_ are dimensionless potentials. This equation can be solved under the conditions of vanishing potentials and fields at the system boundaries. Note that the net charge distribution also contains contributions from the homogeneous rigid background representing the residual charges on the TOCNF. Together with thermal motion, these charges try to avoid a large degree of counterion condensation into the rod’s surface. The density-dependent contribution W̃_*im*_, on the other hand, can be interpreted as an ion-specific interaction that attracts neighboring counterions onto the surface. In the absence of this contribution, eqs 2 and 4 clearly lead to trivial solutions ψ = 0, ρ = *c*_*s*_. Image charge interactions lead to nonuniform potentials close to the interface, and the counterion accumulation onto the surface can be calculated from eq 1. Overall charge neutrality implies in the building-up of an electron layer of opposite magnitude in the inner surface, allowing us to interpret σ_*p*_ as the magnitude of surface charge induced by polarized electrons. Equation 4 can be integrated to provide

5 $$\mathrm{\psi ̃}\left(S\right)=\kappa^{2}\int_{S}^{R}S^{\prime }\ln\left(\frac{S}{S^{\prime }}\right)\left(e^{-\left(\mathrm{\psi ̃}\left(S^{\prime }\right)+{\mathrm{W̃}}_{\mathrm{im}}\left(S^{\prime }\right)\right)}-1\right)dS^{\prime }$$

After a numerical evaluation of eq 3 for given values of *d* and *c*_*s*_, the averaged potential can be readily computed from eq 5 using simple iteration algorithms. The polarizing charge σ_*p*_ is then computed using eq 1. Once this is accomplished, the double layer forces between neighboring CNTs can be estimated by considering the interaction between the two projected surface patches depicted in Figure 1b. At closest surface distances *D* much smaller than the rod diameters *d*, these surfaces can be approximated as two flat, parallel plates of surface charge σ_*p*_, lying at a distance *D* from each other. As before, the intervening region contains mobile counterions of concentration *c*_*s*_, in addition to a uniform background of opposite charge. The disjoint pressure Π between the surfaces can be readily computed as βΠ = ρ­(*D*/2) – *c*_*s*_, where ρ­(*D*/2) is the counterion density at the midplane, where the electrostatic potential is taken to vanish. These quantities can be easily calculated in the Debye–Hückel approximation, which is sufficiently accurate at low surface charges (see SI Material). Once the disjoint pressure Π is obtained, the double layer force between neighboring CNTs in Figure 1b can be estimated by invoking Derjaguin’s approximation^80,88^ to incorporate leading order curvature effects into the flat surface interactions. As a result, the following double-layer force, *F*_DL_, is obtained (see Supporting Information):

6 $${\mathrm{F̃}}_{DL}\left(D\right)=\frac{2\pi \sigma_{p}\lambda_{B}d}{\sin\theta }\ln\left(\frac{\sinh\left(\kappa D/2\right)}{1+\cosh\left(\kappa D/2\right)}\right)$$

where *F̃*_*DL*_(*D*) ≡ βλ_B_ *F*(*D*) defines a dimensionless force, and θ is the aperture angle between the rod axes. The dependence on relative orientations accounts for the increase in magnitude of projecting areas as θ increases. This relation becomes inaccurate for aperture angles such that θ ≤ sin^–1^(2*d*/*L*), which are rather small in situations of high aspect ratios. Note that the force is always repulsive, and features a logarithm divergency at very small separations. As the surfaces are equally charged, the double layer interactions at very close separation are dominated by depletion interactions, as counterions at the overlapping double layers become “loose”, thus increasing mobility and momentum transfer through wall collisions.

Using the same reasoning, the vdW force can be obtained by employing Derjaguin’s approximation,^80^ starting from the interaction between equivalent flat surfaces. The result is

7 $${\mathrm{F̃}}_{vdW}\left(D\right)=-\frac{Ãd\lambda_{B}}{12D^{2}\sin\theta }$$

where again a dimensionless force F̃ is defined via rescaling by a factor βλ_B_, and *Ã* = β*A*, where *A* is the Hamaker constant (throughout this work , the typical value *A* = 10^–19^ J is assigned to this constant). This attractive force has a much smaller range in comparison to double layer forces. However, it displays an algebraic divergency at small separations, which clearly dominates the logarithm behavior observed in eq 6. The balance between these forces is essential to understand the aggregation properties of MWCNTs in solution, as well as the connectedness degree in percolating networks under water uptake.

### 2.7 Pair Connectedness and Percolation Theory

The EDL formation should promote an overall repulsion between the surfaces of neighboring MWCNTs, thereby reducing the amount of closest-distance connections that allow for electron tunneling. In order to quantitatively access these effects on the percolating network, we shall here employ a simple pair connectedness approach, the main aspects of which are highlighted below. Following Hill,^89^ the pair potential *u*(***r***) between fillers is separated into a short-ranged connected *u* ^+^(***r***) and long-ranged disconnected u*­(***r***) contributions, the former being responsible for the formation of short-range bonds that connects neighboring surfaces. In the case of composites, a natural definition of these contributions involves a cutoff distance *r*_*c*_ that delimits the range of action of *u* ^+^(***r***) and u*­(***r***): these quantities are chosen to be ∞ at distances outside and inside these bonds, respectively. Accordingly, the Mayer function *f*(*r*) = *e* ^–β*u*(*r*)^ – 1 is simply split into connected and disconnected parts as *f*(*r*) = *f* ^+^(*r*) + f*­(*r*), where *f* ^+^(*r*) = *e* ^–β*u* ^+^(*r*)^ and f*­(***r***) = *e* ^–βu*(*r*)^ – 1. The functions *f* ^+^(***r***) have their range restricted to the bonding region, and are thus responsible for connecting particles.^90^ Usual *f*-bonds in diagrammatic expansions are turned into a combination of *f* ^+^ and f* bonds. In this formalism, a pair of particles connected by any path made up solely of *f* ^+^ bonds in a cluster diagram are said to be “physically connected” to one another. Sets of diagrams in which any pair of particles are so connected define “physical clusters”, as all particles therein are linked, directly or indirectly, via sets of connecting *f* ^+^ bonds. These clusters are clearly a physical realization of aggregating particles. A cluster which spans the whole sample defines a percolating network.^90^ We begin by considering the situation in which cluster formation is dominated by volume-exclusion repulsive interactions, typical of diluted regimes. In those cases, clustering is dictated solely by geometric and packing constraints.^65^ An order parameter that properly describes averaged cluster sizes in this formalism can be defined as^90^

8 $$\left\langle S^{+}\left(k\to 0\right)\right\rangle =1+\rho \int \left\langle h^{+}\left(r\right)\right\rangle dr$$

where *S* ^+^(*k*) and *h* ^+^(***r***) respectively define the connectedness structure factor and total pair correlation for conducting elements, and the closed brackets $\left\langle \theta \right\rangle \equiv \frac{1}{4\pi }\int d\Omega$ denotes a solid-angle average over orientations of (linear) molecules. Pair connectedness correlations are composed by sums of all diagrams in which every pair of particles is linked by at least one path of *f* ^+^ bonds. The relation above can also be expressed in terms of Fourier components of the direct connectedness pair correlation *ĉ* ^+^(***k***) via the connectedness Ornstein–Zernike (cOZ) relation:

9 $$\left\langle S^{+}\left(k\to 0\right)\right\rangle =\frac{1}{1-\rho \left\langle {ĉ}^{+}(k\to 0\right\rangle }$$

It becomes apparent from this expression that the zeroth wavelength limit ⟨*S* ^+^(*k* → 0)⟩ features an unbound growth as *ρĉ* ^+^(*k* → 0) → 1. Physically, the quantity *S* ^+^(*k* → 0) provides a measure of particle fluctuations, and its divergency indicates the onset of a spinodal decomposition. In percolation theory, it represents the emergence of a fully percolated network: since the cluster spans the whole sample, the range of particle correlations becomes infinitely large. This allows for an unambiguous definition of the percolation threshold as the critical packing fraction ϕ^*c*^ where the condition *S* ^+^(***k*** → 0) → ∞ is satisfied, namely:

10 $$\phi^{c}=\frac{v}{\int \left\langle c^{+}\left(r\right)\right\rangle dr}$$

where *v* is the volume of a single filler. The evaluation of this quantity can be significantly simplified by relying on a leading-order diagrammatic expansion of *c* ^+^(***r***) in terms of *f* ^+^(***r***) bonds, where *c* ^+^(***r***) = *f* ^+^(*r*). We begin by analyzing the case of pure exclusion-volume interactions, where *r*_*c*_ is taken as the overlap distance. In this case, a natural definition of connectedness pair interactions is through the potential well, *u* ^+^(***r***) = 0 for | ***r*** | < *r*_*c*_ and *u* ^+^(***r***) = ∞ otherwise, while u*­(***r***) = *u*(*r*) = ∞ if | ***r*** | < *r*_*c*_ and vanishes otherwise. Hence the connectedness bond *f* ^+^(***r***) = *e* ^–β*u* ^+^(***r***)^ becomes unity at overlapping distances, and vanishes outside this range. The critical packing fraction thus becomes

11 $$\phi^{c}=\frac{v}{\int \left\langle e^{-\beta u^{+}\left(r\right)}\right\rangle dr}=\frac{v}{\left\langle v_{c}\right\rangle }$$

where *v*_*c*_ defines the so-called covolume: the volume which becomes inaccessible to the center of mass of other fillers upon insertion of a given filler in empty space. Note that this quantity is always larger than the particle volume. For the present case of cylindrical insertions, this quantity has been explicitly calculated by Onsager^91^ at various two-particle configurations. We here employ the limiting case of very high aspect ratios *L*/*d*, in which case the covolume becomes^91^ *v*_*c*_ = *L* ^2^d sin θ. Substitution of this result into ([Disp-formula eq11]) and performing the angle average yields the following geometric percolation threshold:

12 $$\phi_{geom}^{c}=\frac{v}{L^{2}d}=\frac{\pi d}{2L}$$

As expected, the leading order contribution for a geometric-induced percolation for slender, rigid cylinders is inversely proportional to the aspect ratio. The role of a geometric constraint in such a dilute regime is rather clear: a large length of an insertion results in a wide region in space which becomes devoid of particle intersection. If insertions are randomly distributed, these geometric constraints mean a large number of close interceptions, such that a small amount of them is sufficient to build-up a percolating network. This situation changes when particle correlations driven by further repulsions are taken into account, as the probability of short-distance intersections become much smaller. In order to quantitatively access these changes in the context of a connectedness percolation theory, we now consider the situation where the cutoff distance corresponds to typical tunneling distances, lying in the range *r*_*c*_ ≈ 0.34 to 1.80 nm.^92,93^ Taken as a generalized coordinate the distance *D* of closest-approach between CNTs surfaces, the potential in Derjaguin’s approximation becomes *u*(***r***) = *u*(*D*)/ sin θ. Since a vdW potential becomes numerically unstable at short separations, we consider a short-range well beyond overlapping, β*u* ^+^(*D*) = – ϵ for 0 < *D* ≤ *r*_*c*_ and β*u* ^+^(*D*) = ∞ if *D* > *r*_*c*_. Our strategy then goes as follows. The attractive potential strength ϵ is first adjusted in such a way that ϕ^*c*^ obtained via eq 10 within a second-virial approximation coincides with the geometric threshold ϕ_geom_^*c*^ given by eq 12. The repulsive contribution resulting from EDL formation is then “turned on” by replacing *u* ^+^(*D*) → *u* ^+^(*D*) + *u*_EDL_(*D*). Equation 10 then allows one to compute a modified critical concentration ϕ_EDL_^*c*^, triggered by surface polarization. A more suitable quantity to measure the relative effects of EDL formation over purely geometric constraints is the ratio ϕ_EDL_^*c*^/ϕ_geom_^*c*^, which can be explicitly written as

13 $$\frac{\phi_{EDL}^{c}}{\phi_{geom}^{c}}=\frac{\int_{0}^{r_{c}+d}\left\langle e^{-\beta \left(u_{EDL}\left(D\right)+u_{0}\left(D\right)\right)/\sin\theta }\right\rangle \mathrm{SdS}}{\int_{0}^{r_{c}+d}\left\langle e^{-\beta u_{0}\left(D\right)/\sin\theta }\right\rangle \mathrm{SdS}}=\langle \exp\left(-\beta u_{EDL}\left(S\right)/\sin\theta \right)\rangle_{0}$$

where the quantity ⟨⟩_0_ is meant to represent an ensemble average over the degrees of freedom of individual insertions in the absence of EDL forces. Note that the averaging is performed over tunneling regions of close surface-to-surface distances, where the Derjaguin’s approximation is expected to be quite accurate. Since EDL forces are always repulsive, addition of these interactions leads to larger filler separations, such that ϕ_EDL_^*c*^ > ϕ_0_^*c*^. Situations where ϕ_EDL_^*c*^/ϕ_0_^*c*^ ≫ 1 clearly indicate that EDL repulsion dominates over random arrangements promoted by swelling (dilution) of the composite.

## 3 Results and Discussions

### 3.1 Experimental Characterization

Representative morphologies of the isolated nanomaterials are shown by atomic force microscopy (AFM) images in Figure 2a,b. TOCNF (a) appears as a dense network of thinner fibrils, with diameters typically below 5 nm. In contrast, multiwalled carbon nanotubes (b) appear as long filaments with diameters ranging from ∼5 to ∼50 nm, and lengths of a few tens of micrometers. In some regions, aggregates reach heights up to 200 nm, indicating incomplete debundling, even in NMP solvent. Both materials exhibit fibril-like morphologies, but with a clear size disparity: the nanofibers are, on average, 1 order of magnitude thinner than the nanotubes.

**Figure 2:**
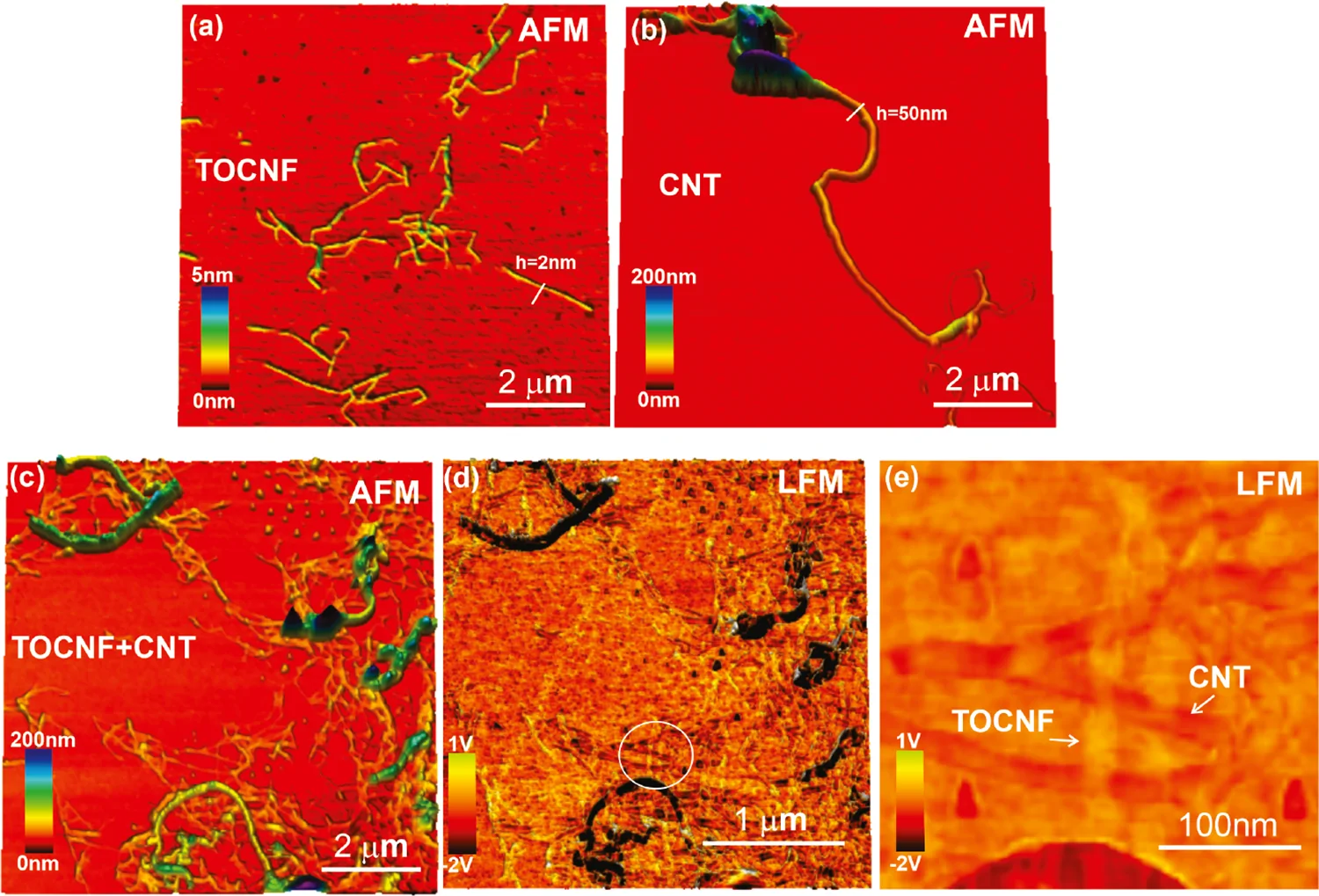
SPM characterization of the base materials and composite ink. (a) TOCNF fibrils in water, forming a network of thinner structures (<5 nm). (b) MWCNTs dispersed in NMP, showing long filaments (5–50 nm in diameter) with partial bundling. (c) AFM image of the diluted composite ink (S2), with CNTs partially surrounded by TOCNF. (d) LFM image of the same region showing different contrast between components: TOCNF in yellow and CNTs in dark brown. (e) Zoomed-in view of the circled region in (d), highlighting these mechanical differences.

These morphological properties (particularly the scale disparity) are particularly useful for the design of sensoring devices triggered by electronic transport, as a small amount of dispersed CNTs is enough to establish a fully connected network through the underlying matrix of small fibers. Such size asymmetries are crucial to understanding carbon nanotubes (CNTs) dispersive ability in aqueous dispersions, as well as our composite’s conductivity response to vapor adsorption, as discussed in what follows.

To evaluate the morphology of the resulting composites after formulation, a diluted aliquot of one hybrid ink (S2) was drop-cast and imaged by AFM and lateral force microscopy (LFM). Figure 2c–e shows a representative region where MWCNTs appear as micrometer-long, elevated filaments, partially surrounded by a dense network of thinner TOCNF fibrils. The morphology suggests that stabilization arises primarily from partial coverage and interposition of TOCNF fibrils between MWCNTs – leading to physical separation and interfacial interactions – rather than a complete coating/encapsulation of the nanotubes.

For the electrical characterization of the inks, we begin by investigating the individual responses of both only-MWCNTs and only-TOCNFs devices to humidity changes. To perform a consistent comparison between the responses of different device compositions, resistance values were obtained through linear fits to the *I* × *V* curves, within ∼−1.5 to ∼+1.5 V (two-probe), i.e., in the near-ohmic regime. Figure 3a,b show representative current–voltage curves of devices fabricated from individual MWCNTs and TOCNFs inks, respectively, under conditions of low and high relative humidity (RH).

**Figure 3:**
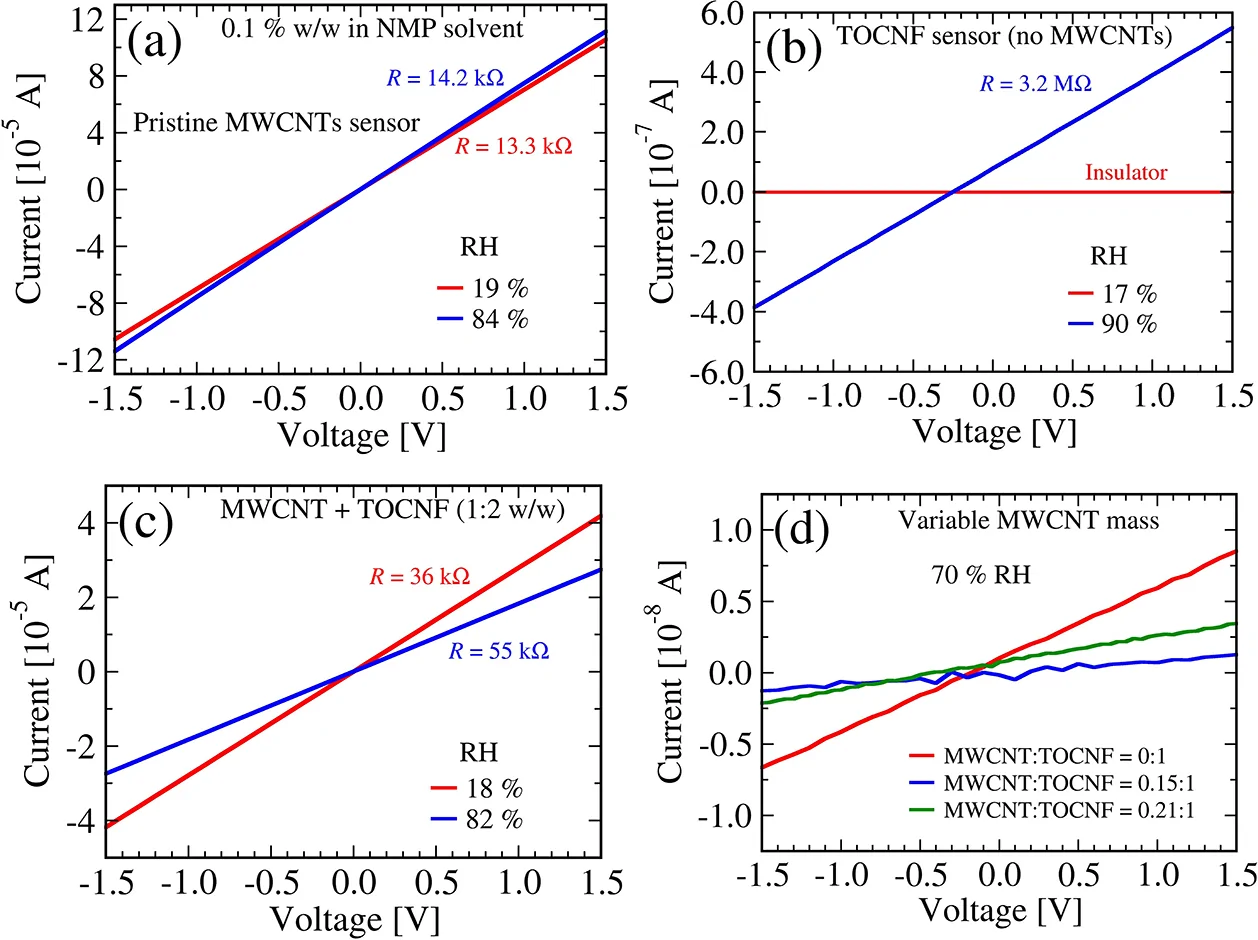
Representative *I* × *V* curves obtained from single devices, at different humidity levels. Plots (a) and (b) show results for sensors based on single components: nonfunctionalized MWCNTs and TOCNF, respectively, at both low and high humidity levels. Panels (c) and (d) show comparisons between humidity responses of MWCNT/TOCNF compositions at different dry mass ratios and humidity levels. In panel (d), the blue and green curves correspond to MWCNT:TOCNF dry-mass ratios of 0.15:1 and 0.21:1, respectively (12.8 and 17.2 wt % MWCNT on a dry-solid basis; nominal dry-solid volume fractions 0.115 and 0.156). Panel (d) contains one device per composition and is used as a qualitative comparison.

The nonfunctionalized MWCNTs-based sensor shows marginal variation with humidity. This reduced response is attributed to two key factors: the lack of hydrophilic functional groups on the nanotube surface, which renders it predominantly hydrophobic character, and the intrinsically high electronic conductivity of percolated MWCNTs networks, which tends to dominate over any minor modulation induced by ambient moisture.^44,45,94,95^

The TOCNF-based sensor, on the other hand, displays a markedly different behavior compared to MWCNTs, with a marked decrease in resistance as humidity increases (Figure 3b). This pronounced response is consistent with an ionic conduction mechanism driven by the dissociation of Na^+^ counterions from carboxylate (−COO^–^Na^+^) groups introduced by TEMPO oxidation. Under dry conditions, TOCNF behaves basically as an insulator due to the absence of a solvent to drive the mobility of charge carriers. However, as the relative humidity increases and more water is adsorbed by the hydrophilic surfaces of the fibers, the high dielectric constant of water promotes the dissociation of Na^+^ counterions from the carboxylate groups, leaving negatively charged residues on the nanofiber backbones, and favoring the formation of hydrated ion transport pathways. The released Na^+^ ions become the dominant mobile species, and their concentration and mobility increase with water uptake. This mechanism has been recently demonstrated experimentally by Zhu et al.,^31^ where it was shown that, above 75% RH, the impedance and capacitance of TOCNF films are primarily governed by the dynamics of Na^+^ and absorbed water. Further investigations have indicated that both surface charge densities and hydration states play crucial roles in controlling ionic conductivity in such systems.^96,97^

A non-negligible shift from the origin observed in the *I*–*V* curve (see Figure 3b) may reflect interfacial effects such as charge condensation or a time-dependent electrode polarization under humid conditions – a behavior not uncommon in ionic conductive nanomaterials measured under DC bias. It is important to highlight the difference in resistance scales observed in Figure 3a,b, which reflects the inherently distinct natures of underlying charge transport mechanisms: electron conduction via MWCNTs channels is about 3 orders of magnitude larger than that of ionic transport through the matrix of functionalized TOCNFs, reflecting the different nature of charge transport, with a much shorter characteristic time-scale for electronic flow. In spite of the larger conductance of the MWCNTs-based device, its sensitivity in detecting changes in ambient moisture is very poor compared to the TOCNFs sensor.

We now proceed to investigate the interplay between the combined effects from MWCNTs/TOCNFs compositions in humidity sensing devices. Figure 3c shows the *I*–*V* behavior for the hybrid ink S3–MWCNT:TOCNF ratio of 1:2.2– at similar levels of humidity changes. A systematic increase in the measured resistances can now be observed by varying the surrounding humidity, in clear contrast to the modest RH dependence of CNT-only devices (Figure 3a) and to the conductance rise observed in TOCNF-only films (Figure 3b). Notably, the baseline resistance of the hybrid is of the same order as that of CNT-only devices, indicating that DC transport is electronically dominated by the percolated MWCNT network rather than by purely ionic pathways in the matrix. This provides a nonsteric route to reduced connectivity that coexists with (but is not reducible to) swelling. Two observations reinforce this view. First, the relatively stable colloidal dispersion of nonfunctionalized MWCNTs in the presence of TOCNF indicates electrostatic interactions strong enough to prevent irreversible aggregation, consistent with double-layer formation that can also operate at solid-state junctions under humidity. Second, SPM characterization reveals long and relatively large-diameter MWCNTs (*L* ∼ 10–30 μm; *D* ∼ 10–30 nm) spanning multiple TOCNF bundles—distinct from systems with shorter CNTs that decorate individual fibers, for which the literature often attributes the conductivity loss to percolation weakening caused by the expansion of the cellulose surfaces.

The asymmetry in *I* × *V* curves observed in Figure 3d, in the absence of a percolated filler network, can be clearly attributed to slow ion relaxation dynamics induced by DC fields. If the field strength changes slowly (in comparison to ionic relaxation times), ion motion under DC fields can be taken as a quasi-adiabatic process. It is well-known that, at sufficiently strong field strengths, significant electrode polarization might take place, eventually suppressing the static surface polarization at the CNTs surfaces. Moreover, “memory” effects resulting from slow decays of correlation dynamics can be present under DC fields, making the ion dynamics dependent on the specific field-changing protocol. It is unclear, however, whether these effects on ion hydrodynamics might influence electronic transport, once a percolating network has been settled.

In order to address this question, we have also performed impedance spectroscopy measurements on films below, close to, and well above the percolation threshold. Figure 4 compares their low-frequency response at approximately 50 and 81% RH. The main contrast is the reversal of the humidity dependence of conductivity across the percolation region (Figure 4a). Increasing humidity raises the apparent conductivity of the below-threshold film, but lowers it in both percolating films. The corresponding median conductance over 20–200 mHz increases by a factor of approximately 8.2 below the threshold and decreases by approximately 32 and 25% near and well above it, respectively. Together with the TOCNF-only response in Figure 3b and the established humidity-dependent ionic response of TOCNF,^31^ this reversal supports a change from predominantly ionic transport through the hydrated matrix to predominantly electronic transport through the connected CNT network. The comparison concerns different compositions; it does not demonstrate a humidity-induced loss of percolation within a single device or separately quantify the two carrier contributions.

**Figure 4:**
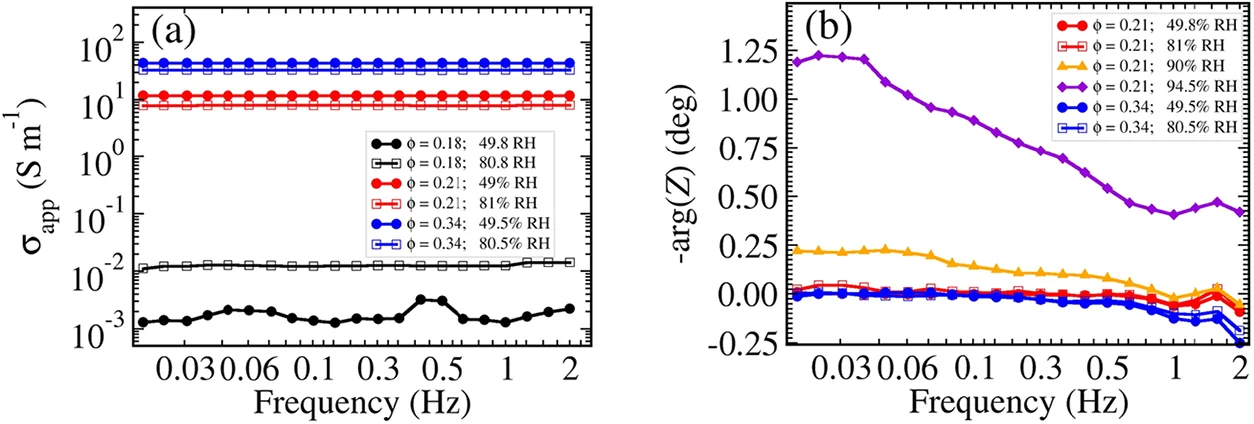
Low-frequency impedance response of MWCNT/TOCNF films. (a) Apparent conductivity of films below (ϕ_*v*_ ≈ 0.18), near (ϕ_*v*_ ≈ 0.21), and well above (ϕ_*v*_ ≈ 0.34) the percolation threshold at approximately 50 and 81% RH. Conductivity uses the common nominal geometry defined in SI. (b) Signed phase, – arg *Z*, of the same near-threshold device at 49, 81, 90, and 94.5% RH, compared with the well-percolated film at 49.5 and 80.5% RH. Positive – arg *Z* denotes capacitive sign. The below-threshold phase and full spectra are provided in the Supporting Material.

In our percolating films, the apparent conductivity is nearly constant at low frequency. Since ε_app_^″^ = σ_app_^′^/(*ε*_0_ω), the apparent dielectric loss increases approximately as 1/ω as frequency decreases (see Supporting Material). Since relaxation times characteristic of electronic transport are orders of magnitude shorter than those of ion motion, electrons are able to collectively rearrange to screen applied fields within a time span where ion motion is negligibly small. This simple scenario is drastically changed in situations of either nonconducting composites or nonpercolating networks, where complex interplays between polymer conformations and ion motion give rise to a rich variety of dielectric responses across characteristic frequency modes.^98–101^ In the present case, our spectroscopy measurements suggest that even a small amount of percolation is enough to suppress such field-induced polarization effects, as electronic responses are far dominant.

We note, however, that some interesting fingerprints of ionic modes can be resolved at very small frequencies and high humidity levels, as shown in Figure 4b. The panel shows that the phase of the percolating films remains close to zero at approximately 50 and 81% RH, consistent with a predominantly resistive response, but when humidity is increased further on the same near-threshold device, a small positive phase becomes apparent at low frequency. The median – arg *Z* over 20–200 mHz reaches approximately 0.21° at 90% RH and 1.02° at 94.5% RH. Thus, a small capacitive contribution becomes detectable while conduction still dominates, possibly indicating a competition starting between ionic and electronic transport close to the onset of a percolation transition. Its humidity dependence is consistent with slow charge redistribution, but the spectra do not identify whether this occurs mainly in the matrix, at CNT interfaces, or at the electrodes. The more scattered phase of the below-threshold film is retained in the Supporting Material and is not used to identify a relaxation mechanism (see SI Material for further details).

It is important to point out that the lack of clear ionic-polarization signals in our spectroscopy data cannot be understood as an absence of counterion polarization at the conducting CNTs interfaces. While the dielectric spectra measures an overall dielectric response to the AC-field, the counterion polarization mechanism resulting from image-charge attractions is an intrinsic interfacial effect, whose manifestation is independent of any external stimuli. Instead, it is triggered by counterion-surface correlations in conductors embedded on an electrolyte,^102–105^ and can lead to intriguing correlation-induced phenomena such as attraction between conducting spheres in bulk electrolytes.^102,106^ Contrary to field-induced polarization, which leads to dipole-like surface charge distributions that oppose the applied field, ion-surface correlations induce the buildup of uniform (monopole) double-layers in virtue of a dielectric mismatch at the interface. Since local fields are usually much stronger than the applied fields (plus the ones they induce) it is reasonable to expect a not too large influence from applied fields on such intrinsic polarization mechanisms.

The steady-state resistance gain, *R*_*G*_ = 100­(*R*_RH_ – *R*_0_)/*R*_0_, was evaluated after normalizing each device to its own low-RH resistance. Figure 5a summarizes the corresponding RH plateaus measured across the independently coated devices. Data represent *n* = 3, except for the two intermediate points, for which *n* = 2.

**Figure 5:**
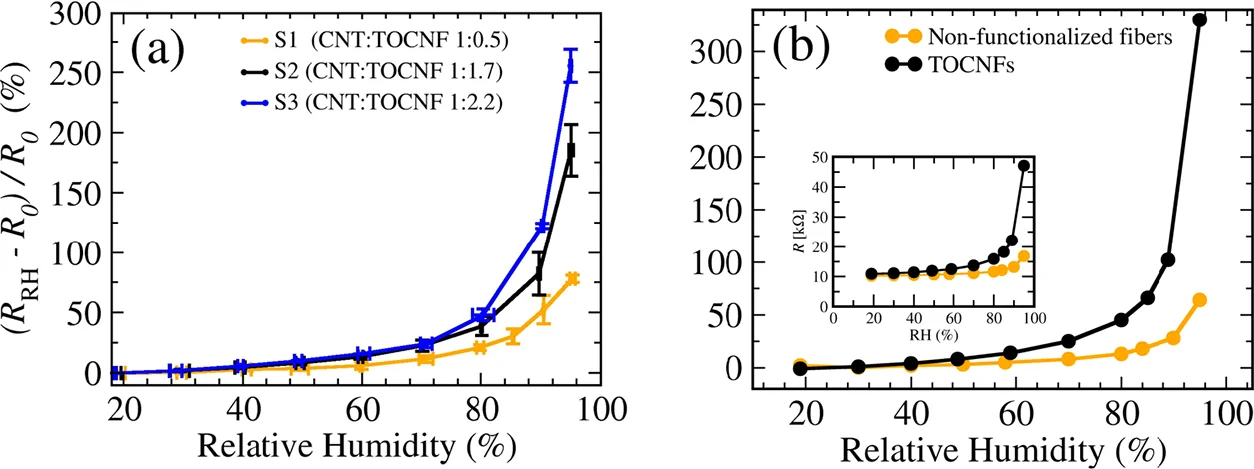
Relative resistance gain as a function of RH. (a) MWCNT/TOCNF devices prepared at the indicated dry-mass ratios. Horizontal and vertical error bars represent one sample standard deviation in RH and resistance gain, respectively. Lines are guides to the eye. (b) Representative single-device comparison between a nonoxidized-fiber composite and the TOCNF composite (*n* = 1 for each control).

The device’s ability of sensing small variations in local humidity can be quantified by the resistance change (relative to its dry state) when subjected to increasing amounts of water vapor. This analysis is performed in Figure 5a, where this quantity is shown as a function of humidity levels for the three representative samples under investigation (see Section 2.3). As discussed in Section 3.1, measurements have been performed following the same set of predetermined protocols.

As shown in Figure 5a, the composite exhibits strong overall performance, with resistance gains reaching up to ∼275% at high relative humidity. Moreover, all devices are clearly able to resolve humidity changes starting from ∼50% RH.The RH-induced resistance gains increase systematically with TOCNF content – the sensors with larger TOCNF concentrations exhibit the most expressive responses at mid- and high-humidity.

Motivated by this trend, we now examine a plausible interfacial route. Our working hypothesis – consistent with the electronic baseline and SPM morphology – relies on the basis of a simple polarization mechanism: as RH increases and Na^+^ counterions are allowed to freely move over the sample through emerging water channels, their image-charge interaction with the conducting MWCNTs (whose exposed area in solution is much higher than that of polymer fibrils) leads to a double layer formation at their surfaces. Concomitantly, the increased mobility of conductive surfaces, “decorated” by polarized counterions, leads to a mutual separation which possibly outweighs the one expected from matrix swelling alone. This view is additionally supported by measurements with Electrical Force Microscopy (EFM) for a S3 ink show that the EFM contrast associated with the MWCNT regions changes upon reversal of the applied bias. The polarity-dependent EFM response can be inferred as experimental evidence for a localized interfacial electrostatic potential that is consistent with the proposed Na^+^ polarized interfacial structure (see SI Material).

Such polarization mechanism entails a large depletion of mobile electrons toward the MWCNT outer surface, thus reducing charge transport driven by an external field. As we shall shortly see, electron surface polarization also leads to an effective repulsion between neighboring MWCNTs, which tends to reduce their nearest-contact area as tube mobility is enhanced in an increasingly wet environment. It is thus likely that the enhanced particle mobility results in a loss of local connections responsible for promoting charge transfer across the percolating network. At a regime of moderate humidity levels (∼50% to ∼ 80%), the degree of water adsorption is large enough to trigger particle mobility, leading to resistance gains that increase moderately as vapor concentration grows. The sample containing the smallest amount of TOCNF shows a lower gain, as both water uptake and the amount of dissolved counterions are smaller at low TOCNF content. As the humidity level grows beyond ∼ 80%, a steep increase in resistance gain is observed in all cases. The devices are thus particularly suitable for detecting tiny humidity changes in this regime.

Vapor penetration into the composite also results in an increase in its overall volume (swelling), effectively reducing the packing fraction of CNTs and hence their overall connectivity. As all samples are expected to be sufficiently wet in a saturation regime of about ∼ 90% RH, it is reasonable to assume that the deviations between samples in this regime can be mostly assigned to distinct amounts of dissolved counterions, rather than to the mechanical swelling of the polymeric matrix. This assumption is further corroborated by the results of Figure 5b, which compare two samples prepared with the same amount of MWCNTs and fibers (dry basis), differing only in fiber functionalization. As the nonfunctionalized nanocellulose lacks dissociable surface groups, the resulting composite contains no dissolved counterions. As a result, the response to ambient humidity is primarily governed by vapor-mediated swelling. As Figure 5b indicates, this effect becomes apparent mainly above ∼ 80% RH. Still, the gains are rather modest (∼50%) compared with the TEMPO-oxidized nanocellulose composite, whose enhanced performance is evident from its much larger gain of ∼330% at high moisture levels. Moreover, a close inspection into the inset of Figure 5b reveals that both TEMPO-functionalized and nonfunctionalized based-composites have quite similar baselines in their dry state. Water adsorption leads to drastically distinct behaviors, particularly in the high-humidity regime where both samples are supposedly fully wet. These results strongly suggest that counterion-mediated interfacial polarization is a major mechanism modulating the conductivity of MWCNT/TOCNF samples under DC bias. This is hardly a direct influence in charge transport (as typical ionic diffusion times in water are way longer than electronic relaxation times in semimetals) but rather an indirect effect that induces electronic localization effects, along with weakly connected networks upon increasingly wet environments.

### 3.2 Model Predictions for Counterion-Induced Polarization

In order to deepen our analysis of the observed experimental findings, we now present some key predictions from the proposed theoretical description (sketched in Section 2.6), which underpin some of the most relevant features controlling charge transport behavior in MWCNT/TOCNF composites. Numerical calculations are presented over a selected range of parameters that best reflect our experimental conditions. Since the emphasis is on a semiquantitative description, no adjustable parameters are employed to fit the experimental data. The numerical solution of eq 5 for an isolated MWCNT surrounded by a background of charged nanofibers and their mobile counterions allows computation of the polarization surface charge density via eq 1 under different MWCNTs sizes and sodium concentrations. This analysis is summarized in Figure 6a,b, which show, respectively, the total volume and surface charge densities of polarization charge on MWCNTs of length *L* = 3 μm and various diameters *d*, resulting from the adsorption of charged counterions onto their surfaces.

**Figure 6:**
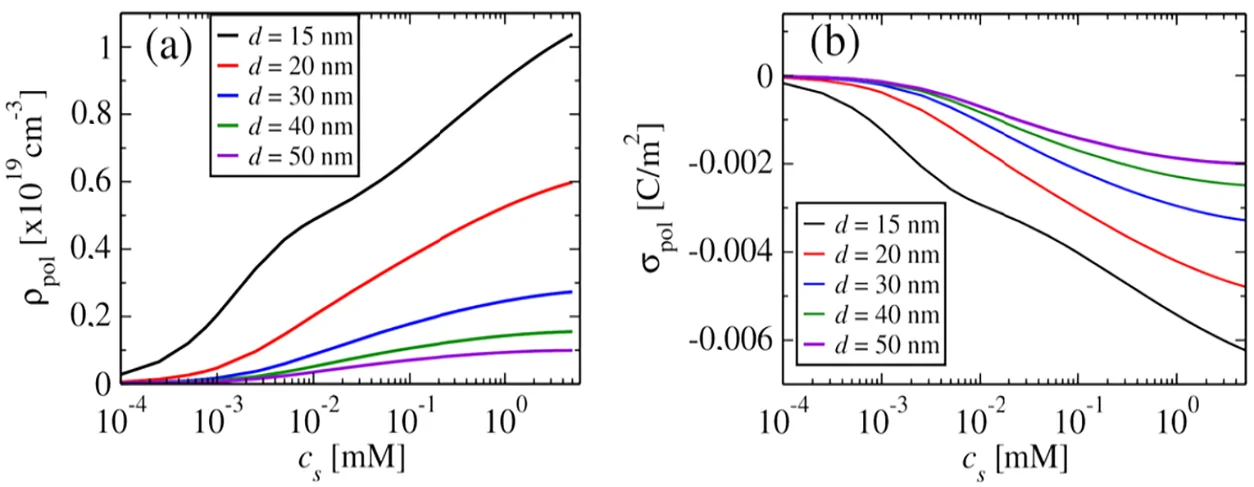
Volume (a) and surface (b) polarization charges induced on MWCNTs in the presence of mobile counterions of concentration *c*_*s*_, resulting from the dissociation of functional groups at the backbones of TOCNF fibers. Predictions are shown for MWCNTs of different diameters *d*, while their length is fixed at *L* = 3 μm.

A substantial polarization charge is induced by the presence of mobile counterions, resulting in an accumulation of charge carriers at the nanotube surface. The strength of such polarizing charges increases monotonically as the concentration of dissolved counterions becomes larger. Such an increase in counterion concentration is meant to emulate situations of either increasing humidity (which promotes counterion release) or higher TOCNF concentrations (which provide more dissociable functional groups). It is interesting to note that the overall density of charge carriers that build up the polarization charge at the MWCNTs interface lies within the range of ∼10^18^ to ∼10^19^ cm^–3^, comparable to charge carrier densities in typical semiconductors (lying between ∼10^16^ to ∼10^19^ cm^–3^). As a result, counterion polarization may reduce the population of charge carriers that effectively contribute to field-driven transport along individual MWCNTs. It is important to note that size polydispersity plays a nontrivial role in particle polarization: CNTs with a smaller surface area (i.e.,larger aspect ratios) feature higher surface densities of polarization charges, irrespective of the sodium concentration *c*_*s*_. This indicates that higher-aspect-ratio MWCNTs tend to repel one another more strongly due to polarization effects.

The surface charge densities in Figure 6, resulting from counterion-induced polarization, can be used to estimate the effective repulsion between neighboring CNTs, as their charged surfaces approach each other. This is readily accomplished by considering the interaction between two parallel plates with fixed surface charges (corresponding to the induced polarization charge on CNTs) at a close separation *D*, as described in the previous section. Derjaguin’s approximation can then be invoked to account for the curvature effects resulting from the combination of electrostatic and van der Waals forces. The resulting forces at small CNT separations *D* are shown in Figure 7 for interactions between nanotubes of equal diameter *d* (additional results for unequal diameters are provided in the Supporting Information), at various sodium concentrations *c*_*s*_.

**Figure 7:**
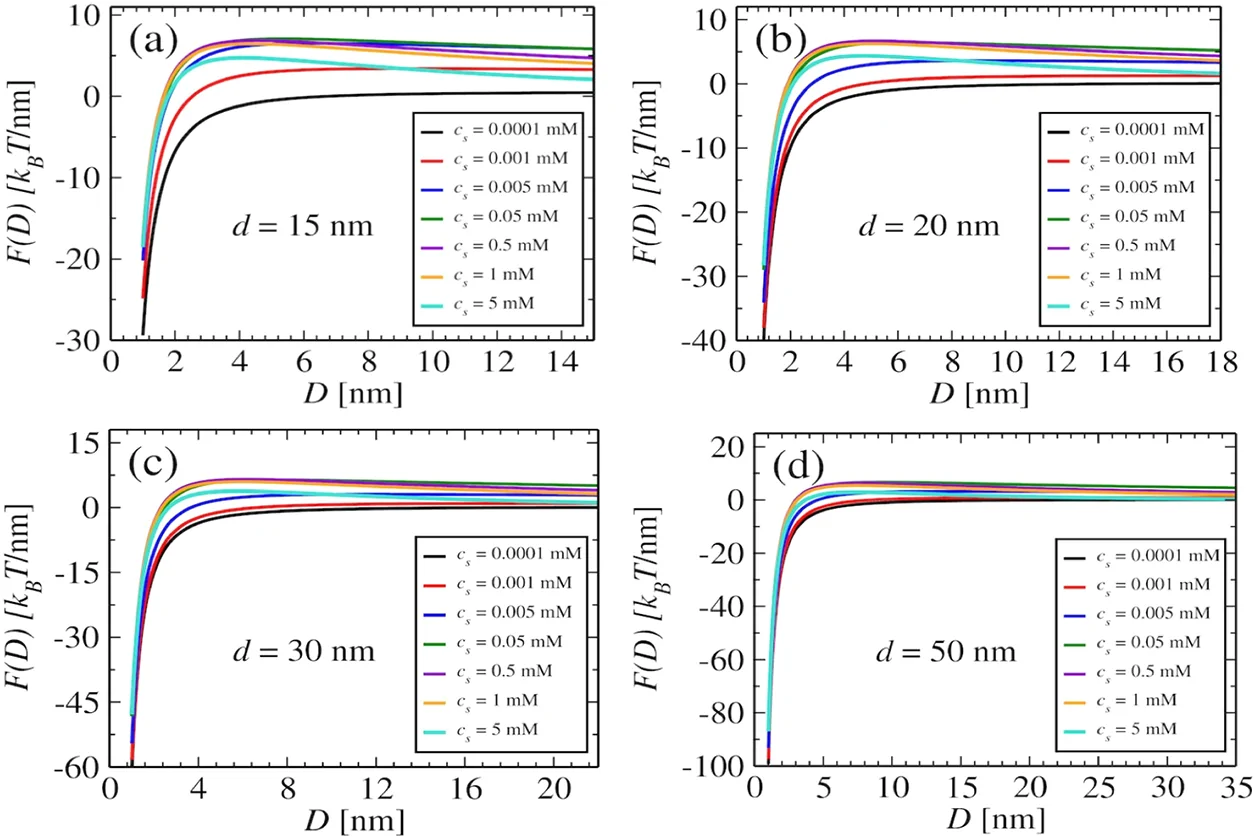
Net forces between the equally sized MWCNTs as a function of the surface-to-surface distance *D*, at several sodium concentrations *c*_*s*_, corresponding to possible achievable sample values upon full dissociation. The particle diameters are (a) *d* = 15 nm, (b) *d* = 20 nm, (c) *d* = 30 nm, and (d) *d* = 50 nm.

At very small counterion concentrations (*c*_*s*_ < 10^–3^ mM) the interactions are purely attractive for all particle sizes and separations. In this regime, van der Waals (vdW) forces dominate over electrostatic interactions arising from particle polarization, and MWCNTs should approach each other as their mobility increases, leading to the formation of permanent bonds that promote charge transfer across the network. As the counterion concentration increases (upon vapor adsorption, for example), the forces undergo a change from attractive (at small distances) to repulsive at larger surface-to-surface separations *D* between nanotubes. Depending on the separation between neighboring CNTs in the initial (dry) state, these particles can either attract or repel one another as the sample becomes wet. In practice, increased particle mobility induces structural rearrangements that slowly attempt to restore the distributions closer to the original (solution) state. The overall effect on macroscopic conductivity is to change the number of effective connections (junctions) that promote charge transfer across the network. This can be accomplished by increasing the distance between CNTs surfaces (via global translation or local bending), or by decreasing their nearest-contact surface area (via CNT reorientations). In either case, the degree of connectedness of the network decreases if neighboring MWCNTs experience mutual repulsion as their mobility increases. Figure 7 reveals a nontrivial interplay between vdW attraction and polarization-induced repulsion: increasing the amount of mobile counterions (*e. g.*, with rising humidity) leads to an effective, charge-mediated repulsion at intermediate surface-to-surface distances (*D* > 3 nm), whose magnitude decreases at larger *D*, eventually giving way to attraction at high counterion concentrations (most likely due to enhanced electrostatic screening). The qualitative force behavior is rather similar for all particle sizes. However, particles with lower aspect ratios (larger surface areas) undergo stronger attraction at close contact, while their mutual repulsion at intermediate distances is slightly larger than for higher-aspect-ratio MWCNTs (despite the larger surface charge induced by counterion polarization; see Figure 6). These results indicate that size polydispersity has nontrivial effects on binding formation between adjacent tubes, beyond the usual exclusion effects considered in classical percolation models.

The combination of short-range attractions and charge-induced, long-ranged repulsions is a common feature in charge-stabilized colloidal systems. Our results strongly suggest that polarization-induced charge stabilization in the presence of TOCNFs counterions is a key mechanism to understand the enhanced dispersion of nonfunctionalized CNTs in solution. The increase in water uptake by the film – driven by humidity uptake at the hydrophilic fibers – induces an overall repulsion of CNTs that tends to recover a colloidal dispersion state. A mechanical equilibrium is established between two neighboring rods at a distance *D*_eq_ where their mutual force vanishes (ignoring thermal fluctuations). This equilibrium distance is shown in Figure 8a, for MWCNTs of different diameters *d*, at various concentrations of dissolved counterions.

**Figure 8:**
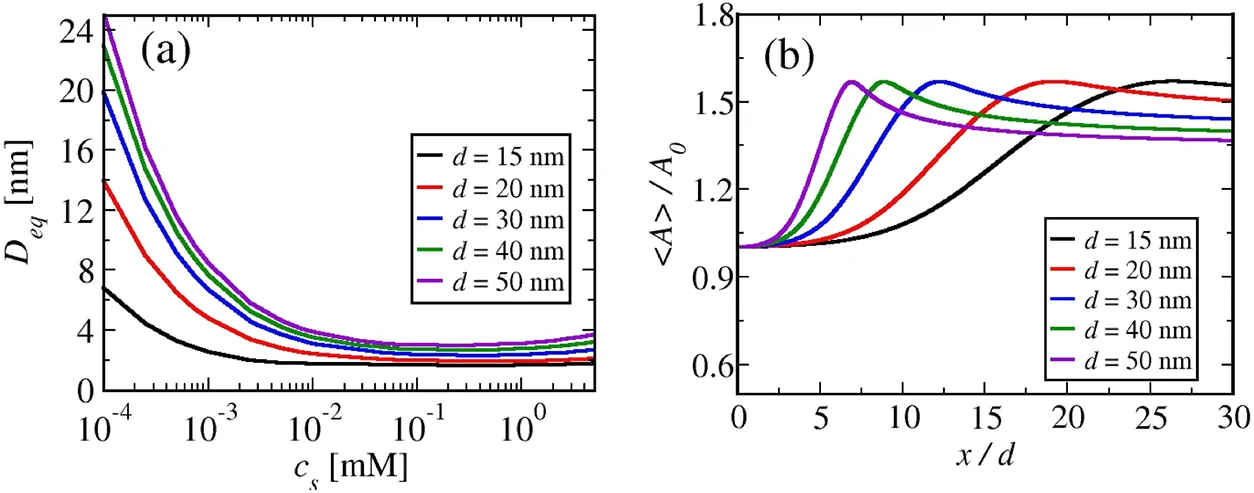
Equilibrium properties of MWCNT embedded into a matrix of fully wet TOCNFs. In (a), the equilibrium separations *D*_*eq*_ of vanishing total force (obtaining by summing up eqs 6 and 7) for differently sized MWCNTs are shown as a function of sodium concentration *c*_*s*_. Panel (b) shows the average mutual exposed area of two neighboring nanotubes, when they are allowed to change their relative orientation at a given distance *x*, at different particle sizes and *c*_*s*_ = 0.5 mM.

At distances *D* < *D*_*eq*_, particles attract each other, tending to form permanent bonds that favor electron tunneling. This range of distances decreases monotonically upon increasing counterion concentration (water vapor adsorption), up to concentrations around ∼ 3 mM. Clearly, the overall effect is to reduce the number of connections in the percolating network, possibly leading to a decrease in overall electronic transport throughout the network. The increase of *D*_*eq*_ at large salt concentrations can be attributed to a stronger screening of electrostatic interactions (small Debye lengths) by the intervening counterions, a well-known effect in colloidal charge stabilization.

Given the large size of the nanotubes under consideration, it is reasonable to expect that increased mobility will manifest more strongly via particle reorientation rather than translation of their centers of mass. Using the proposed approach, it is possible to estimate the average mutual exposed area ⟨*A*⟩ between two neighboring nanotubes (see Figure 1) in the limit of large dilutions, when they are allowed to change their relative orientation (further details can be found in the Supporting Information). This is illustrated in Figure 8b, which shows the ratio between the averaged exposed area, ⟨*A*⟩, and its smallest value *A*_0_ = *d* ^2^ (corresponding to perpendicularly oriented rods) as a function of the surface distance *D*, for a sodium concentration *c*_*s*_ = 0.5 mM (similar results at different concentrations are shown in Supporting Information). At all particle sizes, the average mutual area at very close separations (*D* ≈ *d*) is close to its minimum value, (⟨*A*⟩/*A*_0_ ≈ 1). Due to the dominant double layer repulsion between the surfaces, adjacent rods will rotate to a ∼ 90° angle in an attempt to minimize their mutual area facing each other. This clearly results in a loss of local connections along the network, thus reducing the probability of electronic hopping necessary for charge transport. As the distance between the surfaces increases, the reduced repulsion leads to an increasing alignment between the rods, and a corresponding increase in their exposed area. The area then reaches a maximum value at intermediate distances, before relaxing to an equilibrium value ⟨*A*⟩/*A*_0_ ≈ π/2 at large distances, corresponding to fully random orientations between the rod’s axes.

Further insights on the effects of inferred loss in filler’s connections can be obtained by combining the EDL repulsion with simple pair connectedness calculations outlined in Section 2.7. The main results of this analysis are presented in Figure 9, which shows the shift in percolation thresholds (see eq 13) upon addition of EDL forces in a system of otherwise randomly distributed fillers in dilute regimes. Two cutoff distances for surface separation *D* have been employed: *r*_*c*_ = 0.9 nm (solid lines) and *r*_*c*_ = 1.8 nm (dashed curves), both lying within the range of intersurface distances of non-negligible electron tunneling probability. This criterion is important, as it guarantees that any pair of conducting fillers belonging to a same cluster are always able to exchange their charge carriers through a set of (physically conceivable) tunneling connections. As Figure 9 clearly shows, quite significant deviations in percolation thresholds can be induced by the presence of a charge-polarization mechanism. The high ratios ϕ_EDL_^*c*^/ϕ_geom_^*c*^ at intermediate salt concentrations clearly indicate polarization-induced loss in connectivity: the strong repulsion between polarized surfaces prevents the building-up of large connected clusters, thus requiring a much higher packing fraction of fillers for the assembling large clusters. The large thresholds shifts in Figure 9 clearly decouple the effects of double-layer repulsions from those of matrix swelling driven by vapor adsorption. If EDL repulsions were absent, water intake would have an overall effect of increasing the effective volume available for fillers to rearrange. Weak surface correlations would lead to filler distributions resembling that of random insertions whose connectivity is mostly dictated by geometric constraints, and the percolation threshold would increasingly approach ϕ_geom_^*c*^ upon large dilutions. It is also important to note that the ratios in Figure 9 are little to the choice of cutoff criterion, especially at large surface diameters. Moreover, the shift is larger at smaller diameters, clearly due to the larger charge densities observed in Figure 8b. Another interesting feature is the nonmonotonic behavior of ϕ_EDL_^*c*^/ϕ_geom_^*c*^ observed in all cases, where a maximum ratio is reached at intermediate counterion concentrations (whose range agrees with the typical values estimated from the TOCNF concentrations of our sample, upon full dissociation). At very small *c*_*s*_, both polarization and depletion effects are not strong enough to provide significant EDL repulsions. Increasing *c*_*s*_ enhances polarization effects, leading to pronounced, polarization-induced repulsions that increase ϕ_EDL_^*c*^. Finally, further increase of *c*_*s*_ eventually triggers a significant degree of charge screening, leading to the observed decrease ϕ_EDL_^*c*^ at higher counterion concentrations.

**Figure 9:**
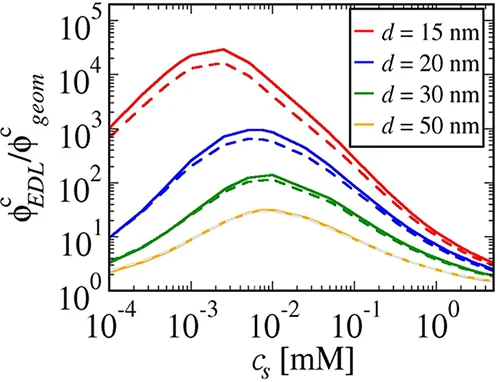
Ratios between EDL- and geometric-induced percolation thresholds calculated via eq 13, as functions of counterion concentrations *c*_*s*_. Dashed lines represent ratios using a cutoff distance connectedness criterion of *r*_*c*_ = 0.9 nm, whereas solid lines are obtained using *r*_*c*_ = 1.8 nm. In all cases, the CNT lengths are fixed at *L* = 3 μm. In the case *d* = 50 nm, dashed lines are displayed in gray color to clearly distinguish it from the nearby orange solid line.

## 4 Conclusions

This study elucidates the interfacial mechanisms governing mixed ionic-electronic transport in MWCNT/TOCNF composites under humidity, while demonstrating large humidity-induced resistance gains (up to approximately 270% in the MWCNT/TOCNF composites). In contrast to most humidity-responsive CNT/polymer systems based on synthetic conducting polymers or functionalized CNTs, our work uses TOCNF to efficiently disperse nonfunctionalized MWCNTs into homogeneous, sprayable inks, providing a water-processable route that avoids CNT functionalization. MWCNT-only films are essentially RH-insensitive, TOCNF-only films display ionic behavior, and TOCNF-rich composites show the most pronounced RH-driven gains – consistent with an interfacial contribution involving mobile counterions that coexists with, but cannot be reduced to, matrix swelling. A control based on nonoxidized nanocellulose (lacking dissociable counterions) exhibits only modest increases at high RH, supporting the contribution of TOCNF carboxylate groups and their counterions to the modulation observed with TOCNF.

For the percolating films, the ionic effects are indirect: the measured currents are predominantly carried by the connected MWCNT network rather than by a standalone ionic pathway. To interpret the modulation, we proposed a polarization mechanism in which mobile Na^+^ counterions, increasingly dissociated and mobilized upon hydration, interact with MWCNT interfaces via image-charge interactions. A theoretical model was then designed to provide qualitative estimations on the strengths of these surface charges, as well as on the resulting CNT–CNT surface-to-surface interactions at close contact, under counterion concentrations and CNT sizes lying within the range of experimental parameters. We find that a considerable amount of charge carriers – whose densities can approach typical semiconductor values – are polarized by surrounding counterions. Moreover, the induced electrostatic repulsion between MWCNTs can be large enough to compete with (and at intermediate distances overcome) van der Waals attraction. Such repulsions could increase the effective separation between neighboring MWCNTs when water uptake enhances nanotube mobility in the film, thereby decreasing the probability of electron tunneling required to maintain charge transport through the network. Further validation of such indirect, EDL-mediated effects on network’s connectivity was provided by calculations based on pair connectedness correlations, performed in the context of a second-virial approximation. These calculations show that EDL repulsions might lead to significant shifts in percolation thresholds with respect to the purely packing and geometric constraints that dominate cluster formation upon dilution of the network. They thus provide a clear indication of a nontrivial role played by counterion polarization in the loss in network connectivity.

The composition series and impedance measurements distinguish transport regimes on opposite sides of the percolation threshold. Humidity increases the low-frequency conductance below percolation but decreases it in connected CNT networks, indicating a change in the dominant transport pathway across the percolation region. In the percolating films, the weak frequency dependence of the low-frequency conductivity is consistent with predominantly electronic transport through the CNT network. Below percolation, the humidity-induced increase in conductivity and the stronger low-frequency reactive response indicate an increased relative contribution from ionic transport and polarization processes. The AC measurements therefore support the participation of mobile charge but do not, by themselves, establish the microscopic distribution of counterions at the CNT interfaces. Together with the composition-dependent DC response, the nanocellulose controls, and the equilibrium model, these results support a counterion-mediated interfacial contribution to the modulation of CNT junction connectivity while neither excluding matrix swelling nor claiming direct microscopic proof of the proposed ion distribution.

The steady-state response was reproduced across independently coated devices, and consecutive RH cycles demonstrated reversible behavior after an initial conditioning cycle during the experimental session. These results establish short-term operational stability and device-to-device reproducibility of the steady-state response, although extended aging and broader dynamic statistics remain subjects for future investigation. Further systematic variation of composition near the percolation threshold, ionic strength, counterion valence, and pH should help separate the respective roles of network connectivity, swelling, and interfacial electrostatics. Combining these experiments with polarization and percolation models may provide a rational framework for designing humidity-responsive materials based on charged nanocellulose and nonfunctionalized CNTs.

This strategy can be used to design humidity-responsive devices with extraordinary performance. Methodologically, complementary measurements such as impedance spectroscopy, systematic control of ionic strength/valence/pH, and microfluidic/flow-through operation could enable robust, quantitative sensing in aqueous media based on this phenomenology.

## Supplementary Material



## Notes

Coarse-Graining Interaction Model and Detailed Experimental Conditions (PDF)
