# Supplementary material for: Counterion-Mediated Humidity Modulation of Electronic Transport in Pristine MWCNT/TOCNF Composites
Source: ACS Omega. 2026 Oct 2;11(40):60831–47. doi: 10.1021/acsomega.6c04065 (PMC13670278; doi:10.1021/acsomega.6c04065)
Supplement: Supplementary file 1 [file ao6c04065_si_001.pdf]

Supplementary Theoretical and Experimental Information Material - Article: Counterion-Mediated Humidity Modulation of Electronic Transport in Pristine MWCNT/TOCNF Composites

Andreza J. Siqueira,[1, a)] Wflander M. Souza,[1, a)] Rayssa O. Matos,[1] Hana H. Koga,[2] Vinicius Ornelas,[3] Claudio Laudares,[3] Dalila C. Sicupira,[2] Thiago E. Colla,[1] Ive Silvestre,[1] and Ana Paula M. Barboza[1, b)]

[1)] *Department of Physics, Universidade Federal de Ouro Preto, Ouro Preto – MG, Brazil*

[2)] *Department of Chemistry, Universidade Federal de Ouro Preto, Ouro Preto – MG, Brazil*

[3)] *CTNano, Department of Physics, Universidade Federal de Minas Gerais, Belo Horizonte - MG, Brazil*

[a)] These authors contributed equally to this work

[b)] Corresponding author; Electronic mail: ana.barboza@ufop.edu.br

I. *COARSE GRAINING INTERACTIONS BETWEEN MWCNT MEDIATED BY MOBILE COUNTERIONS IN HIGH HUMIDITY REGIMES.*

Much of the physics behind the observed conductivity response of our sensor device upon changes of humidity and matrix/CNT concentrations can be rationalized in terms of simple mechanisms described by macroscopic electrostatics, as we demonstrate below. To shed light on these mechanisms, let us first consider the rather simplified picture of the polarization response induced by an isolated Na ion in the close vicinity of a CNT, embedded on an aqueous environment, as sketch in Fig. 1. The ion is modeled as a point charge e, whereas the nanotube is considered to be an infinitely long rod of radius a, whose symmetry axis lies along the z direction. The dielectric constants inside and outside the rod are denoted as ε_1 and ε_2, respectively. Apart from the test charge, the system also contains free mobile ions dissolved from a polymeric matrix. Insertion of a test particle into the electrolyte leads to two distinct polarization mechanisms: an ion cloud of opposite charge is developed around the test particle, in an attempt to screen its field. The second mechanism refers to a polarizing charge at the rod surface, which results from the dielectric mismatch across the interface. Since $\varepsilon_1 > \varepsilon_2$, the net polarizing charge is negative, and tries to screen the field inside the rod. Alternatively, such a polarizing charge can be represented as a set of image-charges induced inside the rod in such a way as to render the displacement field constant across the interface. To investigate the interplay between these polarizing mechanisms, let us consider that the point charge is placed at a distance $S' > a$ from the center of the tube. The induced potential $G(\boldsymbol{r}, \boldsymbol{r}')$ at

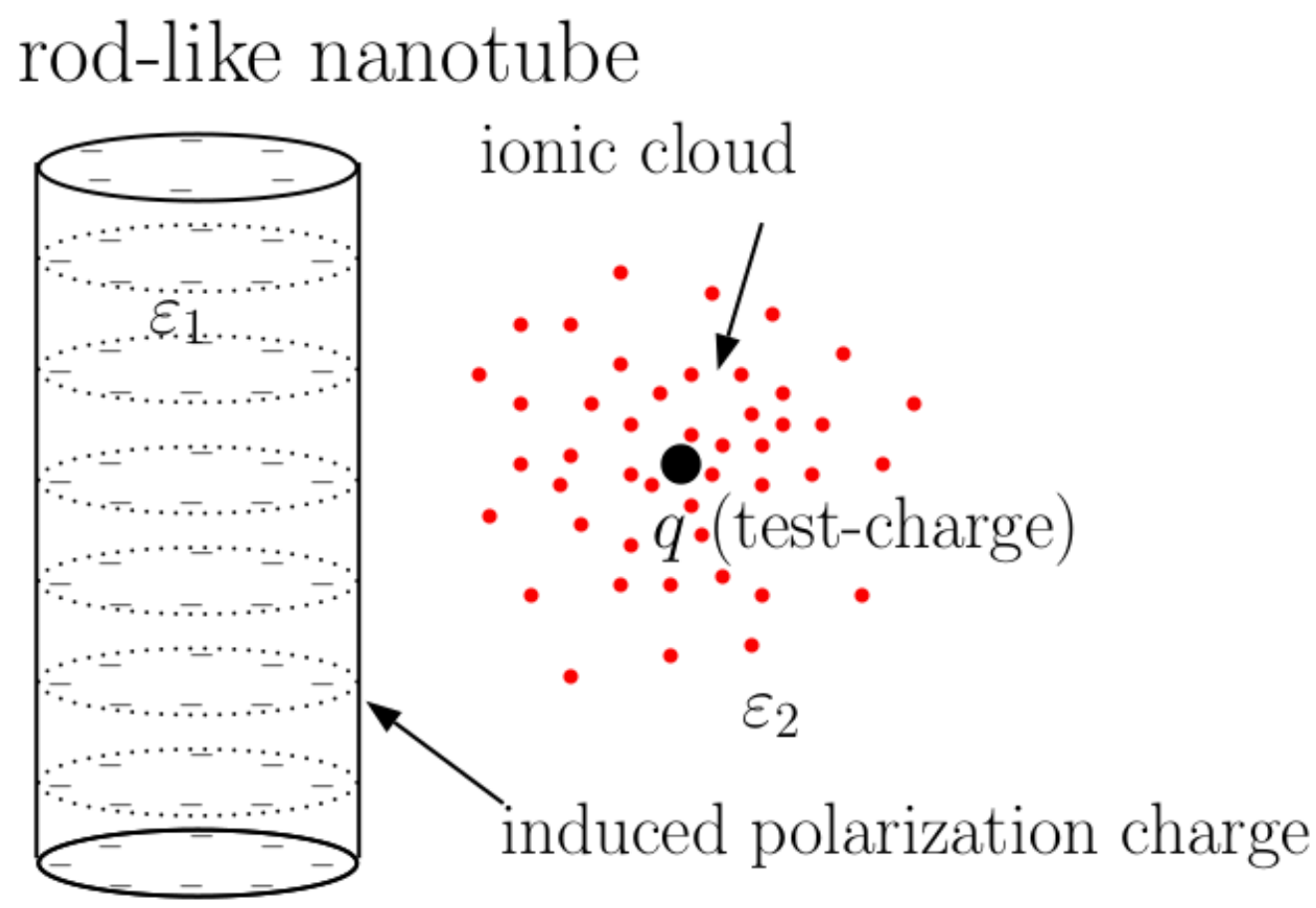

FIG. 1. Sketch of our polarizing model system: a point charge of magnitude e is placed in the close vicinity of a rod-like nanotube of internal permittivity ε_1. The system is embedded into a wet environment of permittivity ε_2, with residual charges that screen the field produced by the test-charge.

a position $\boldsymbol{r} = S\hat{\boldsymbol{e}}_s + z\hat{\boldsymbol{e}}_z$ must satisfy the following Green function equation

$$(\nabla^2 - \kappa^2)G(\boldsymbol{r}, \boldsymbol{r}') = -\frac{4\pi e}{\varepsilon_2}\delta(\boldsymbol{r} - \boldsymbol{r}'), \tag{1}$$

for positions $S > a$, where κ stands for the inverse Debye screening length of the dissolved electrolyte. This quantity is responsible for the screening of the inserted charge by the intervening mobile charges. Inside the tube, $S < a$, the Green function should satisfy the Laplace equation, $\nabla^2 G(\boldsymbol{r}, \boldsymbol{r}') = 0$. In the region $S > a$, the quantity $G(\boldsymbol{r}, \boldsymbol{r}')$ can be readily identified as the Green function for the Helmholtz operator. In cylindrical coordinates, Eq. (1) can be written as

$$\left[\frac{1}{S}\frac{\partial}{\partial S}\left(S\frac{\partial}{S}\right) + \frac{1}{S^2}\frac{\partial^2}{\partial \varphi^2} + \frac{\partial^2}{\partial z^2} - \kappa^2\right] G(\boldsymbol{r}, \boldsymbol{r}') = -\frac{4\pi e}{\varepsilon_2 S}\delta(S - S')\delta(z)\delta(\varphi). \tag{2}$$

Note that we have set $z' = 0$ and $\varphi' = 0$, a choice which is possible due to both rotation and translation symmetries with respect to the cylinder's axis. It is convenient to expand the Green function in term of its Fourier components $g_m(S, S', k)$ along azimuthal and longitudinal coordinates as

$$G(S, S', \varphi, z) = \frac{1}{(2\pi)^2} \sum_{m=-\infty}^{\infty} e^{im\varphi} \int_{-\infty}^{\infty} g_m(S, S'; k) e^{ikz} dk. \tag{3}$$

Accordingly, the delta functions along z and φ coordinates in (2) can be respectively expanded as

$$\delta(z) = \frac{1}{2\pi} \int_{-\infty}^{\infty} e^{ikz} dk, \tag{4}$$

$$\delta(\varphi) = \frac{1}{2\pi} \sum_{m=-\infty}^{\infty} e^{im\varphi}. \tag{5}$$

Substitution of the expansions (3), (4), and (5) into Eq. (2) leads to the following 1D Green function equation for the coefficients $g_m(S, S'; k)$:

$$\frac{\partial^2 g_m(S, S'; k)}{\partial S^2} + \frac{1}{S} \frac{\partial g_m(S, S'; k)}{\partial S} - \left(\frac{m^2}{S^2} + q^2 \right) g_m(S, S'; k) = -\frac{4\pi e}{\varepsilon_2 S} \delta(S - S'), \tag{6}$$

for $S > S'$, where $q \equiv \sqrt{k^2 + \kappa^2}$. Since the interior of the tube ($S < a$) contains neither dissolved electrolyte nor sources, the coefficients in this region are bound to satisfy

$$\frac{\partial^2 g_m(S, S'; k)}{\partial S^2} + \frac{1}{S} \frac{\partial g_m(S, S'; k)}{\partial S} - k^2 g_m(S, S'; z) = 0. \tag{7}$$

The coefficients $g_m(S, S'; k)$ can thus be identified with the 1D Green function for the modified Bessel equation with a source at S' and an interface at $S = a$. Since

the tube interface contains no free charges, solutions to Eqs. (6) and (7) should be linked at the interface $S = a$ *via* the conditions of continuity of both potential and electric displacement,

$$g_m^{(I)}(S = a, S'; k) = g_m^{(II)}(S = a, S'; k), \tag{8}$$

$$\varepsilon_1 \frac{dg_m^{(I)}}{dS}\bigg|_{S=a} = \varepsilon_2 \frac{dg_m^{(II)}}{dS}\bigg|_{S=a}, \tag{9}$$

where $g_m^{(I)}$, $g_m^{(II)}$ and $g_m^{(III)}$ represent solutions in regions $S < a$, $a < S < S'$, and $S > S'$, respectively. Moreover, solutions of Eq. (6) at regions $S > a$ should satisfy the following conditions at the source point ($S = S'$):

$$g_m^{(II)}(S = S', S'; k) = g_m^{(III)}(S = S', S'; k) \tag{10}$$

$$\frac{\partial g_m^{(III)}}{\partial S}\bigg|_{S=S'} - \frac{\partial g_m^{(II)}}{\partial S}\bigg|_{S=S'} = -\frac{4\pi e}{\varepsilon_2 S'}. \tag{11}$$

The second condition above follows directly from integration of Eq. (6) across the close vicinity of the source point, $S = S'$. Apart from these conditions, the coefficients $g_m(S, S'; k)$ should be bound everywhere. Solutions to Eqs. (6) and (7) satisfying such conditions can be generally written as

$$g_m^{(I)}(S, S'; k) = A_m(k) I_m(kS) \tag{12}$$

$$g_m^{(II)}(S, S'; k) = B_m(k) I_m(qS) + C_m(k) K_m(qS) \tag{13}$$

$$g_m^{(III)}(S, S'; k) = D_m(k) K_m(qS), \tag{14}$$

where $I_m(x)$ and $K_m(x)$ represent the mth-order modified Bessel functions of first and second kinds, respectively. The unknown coefficients $A_m(k)$, $B_m(k)$, $C_m(k)$ and

$D_m(k)$ can be uniquely determined using the boundary conditions (8), (9), (10), and (11). After some straightforward algebraic manipulations, we find

$$B_m = \frac{4\pi e K_m(qS')}{\mathcal{W}_m(kS')}$$

$$A_m = -\frac{\varepsilon_1 q \mathcal{W}_m(qa) B_m}{\varepsilon_1 q K'_m(qa) I_m(ka) - \varepsilon_2 k I'_m(ka) K_m(qa)} \tag{15}$$

$$C_m = \frac{\varepsilon_2 k I'_m(ka) I_m(qa) - \varepsilon_1 q I'_m(qa) I_m(ka)}{\varepsilon_1 q K'_m(qa) I_m(qa) - \varepsilon_2 k I'_m(ka) K_m(qa)} B_m \tag{16}$$

$$D_m = \left[\frac{I_m(qS')}{K_m(qS')} + \frac{\varepsilon_2 k I'_m(ka) I_m(qa) - \varepsilon_1 q I'_m(qa) I_m(ka)}{\varepsilon_1 q K'_m(qa) I_m(qa) - \varepsilon_2 k I'_m(ka) K_m(qa)}\right] B_m, \tag{17}$$

where $\mathcal{W}_m(x) \equiv I_m(x)K'_m(x) - I'_m(x)K_m(x)$ is the Wronskian of Eq. (7) (with the prime denoting a derivative with respect to the function's argument). It follows from Eq. (7) that this quantity can be evaluated as $\mathcal{W}_m(x) = E_m/x = 1/x$ [a] Substitution of these coefficients into Eqs. (12), (13) and (14) lead to the following solution for the 1D coefficients:

$$g_m^{(I)}(S, S'; k) = \frac{4\pi e K_m(qS') I_m(kS)}{qS'^2} \left[\frac{I'_m(qa)K_m(qa) - K'_m(qa)I_m(qa)}{\varepsilon_2 q K'_m(qa) I_m(ka) - \varepsilon_1 k K_m(qa) I'_m(ka)}\right], \tag{18}$$

$$g_m^{(II)}(S, S'; k) = \frac{4\pi e K_m(qS')}{\varepsilon_2 q^2 S'^2} \left[\frac{\varepsilon_1 k I_m(qa) I'_m(ka) - \varepsilon_2 q I'_m(qa) I_m(ka)}{\varepsilon_1 k K_m(qa) I'_m(ka) - q\varepsilon_2 K'_m(qa) I_m(ka)} K_m(qS) - I_m(qS)\right],$$

$$g_m^{(III)}(S, S'; k) = \frac{4\pi e K_m(qS)}{\varepsilon_2 q^2 S'^2} \left[\frac{\varepsilon_1 k I_m(qa) I'_m(ka) - \varepsilon_2 q I'_m(qa) I_m(ka)}{\varepsilon_1 k K_m(qa) I'_m(ka) - q\varepsilon_2 K'_m(qa) I_m(ka)} K_m(qS') - I_m(qS')\right].$$

Note that, in the limit of an ideal metallic rod, $\varepsilon_1 \to \infty$, the coefficients in Eq. (19) vanish, and the electric field from the external source is fully screened at the

[a] The integration constant $E_m = 1$ can be obtained from the well-known limiting laws $I_m(x) \sim \frac{x^m}{2^m m!}$ and $K_m(x) \sim \frac{2^{m-1}(m-1)!}{x^m}$, as $x \to 0$.

metal interface. The overall potentials can be obtained upon substitution of the above coefficients into Eq. (3). The resulting potential can be split into two main contributions: the electrostatic potential produced by the test charge (screened by the surrounding ionic cloud), in addition to the potential produced by the interface in response to an external field on its vicinity. This latter quantity defines the so-called *reaction potential*, induced by the surface in an attempt to minimize the electrostatic energy on its interior. This contribution is the one responsible for the polarization charge on the tube's surface. In order to evaluate the reaction potential, one should simply subtract from the coefficients above the contributions from the ionic potential in the absence of an interface (*i. e.*, in a bulk electrolyte), before plugging them into Eq. (3). The contributions produced by the ion in the absence of an interface can be readily evaluated as

$$g_m^b(S,S';k) = \lim_{\substack{\varepsilon_1\to\varepsilon_2 \\ k\to q}} g_m(S,S';k) = \begin{cases} -\dfrac{4\pi e K_m(qS')I_m(qS)}{\varepsilon_2 q^2 S'^2}, & S < S', \\ \\ -\dfrac{4\pi e K_m(qS)I_m(qS')}{\varepsilon_2 q^2 S'^2}, & S > S'. \end{cases} \tag{19}$$

Here, the superscript b reminds us that the coefficients stand for an isolated ion placed into a bulk electrolyte. Substitution of this equation into (3) recovers the well-known Debye-Hückel screened potential of a point-like ion, $G(\boldsymbol{r},\boldsymbol{r}') = \dfrac{e}{\varepsilon_2}\dfrac{e^{-\kappa|\boldsymbol{r}-\boldsymbol{r}'|}}{|\boldsymbol{r}-\boldsymbol{r}'|}$. Now, the Fourier coefficients of the reaction potential, $g_m^{reac}(S,S';k)$ can be computed by subtracting Eq. (19) from the coefficients in (18). The resulting coefficients in

the case $S < a$ read as

$$g_m^{reac}(S, S'; k) = \frac{4\pi e K_m(qS')}{\varepsilon_2 q^2 S'^2} \left[\frac{\varepsilon_2 q I_m'(qa) K_m(qa) - \varepsilon_2 q I_m(qa) K_m'(qa)}{\varepsilon_2 q K_m'(qa) I_m(ka) - \varepsilon_1 k K_m(qa) I_m'(ka)} I_m(kS) + I_m(qS) \right], \tag{20}$$

whereas in the region outside the surface $S > a$ they can be written as

$$g_m^{reac}(S, S'; k) = \frac{4\pi e K_m(qS) K_m(qS')}{\varepsilon_2 q^2 S'^2} \left[\frac{\varepsilon_2 q I_m(ka) I_m'(qa) - \varepsilon_1 k I_m(qa) I_m'(ka)}{\varepsilon_2 q K_m'(qa) I_m(ka) - \varepsilon_1 k K_m(qa) I_m'(ka)} \right]. \tag{21}$$

Notice that the reaction potential is now continuous across the source charge, as it represents the induced potential due to the interface, in response to a nearby source. However, the reaction field features a discontinuity across the interface, which can be interpreted as a polarizing surface charge induced the dielectric mismatch. From Eqs. (20) and (21), the discontinuity "jump" can be readily evaluated as

$$\left. \frac{\partial g_m^{reac}(S, S'; k)}{\partial S} \right|_{S=a_+} - \left. \frac{\partial g_m^{reac}(S, S'; k)}{\partial S} \right|_{S=a_-} = \frac{4\pi e k I_m'(ka) K_m'(qS')(\varepsilon_2 - \varepsilon_1)}{\varepsilon_2 a} \left[\frac{1}{\varepsilon_2 q K_m'(qa) I_m(ka) - \varepsilon_1 k K_m(qa) I_m'(ka)} \right]. \tag{22}$$

Here, the relation $\mathcal{W}_m(qa) = I_m'(qa) K_m(qa) - I_m(qa) K_m'(qa) = 1/qa$ for the Wronskian was used once again. By virtue of Gauss law, the polarizing charge at the rod's surface can be obtained as $\sigma_{pol} = -\varepsilon_0 \left(\nabla\psi_+ - \nabla\psi_-\right) \cdot \hat{\boldsymbol{e}}_s / 4\pi$, where ψ_+ and ψ_- are the potentials just after and before the surface is crossed, respectively. For a fixed distance S' between the test charge and the center of the rod, the induced polarizing surface charge can thus be computed upon insertion of this expression into Eq. (3). However, it proves more instructive to calculate the total induced surface charge

within an angle aperture $d\varphi$ around φ. Let $f(\varphi)$ be the ratio between the enclosed charge dQ_{pol} and $d\varphi$. This quantity can be computed upon substitution of Eq. (22) into (3), followed by an integration over z. Using Eq. (4), the result is

$$f(\varphi) = e\left(\frac{\varepsilon_1}{\varepsilon_2} - 1\right) \sum_{m=-\infty}^{\infty} e^{im\varphi} \lim_{k\to 0} \left[\frac{kI'_m(ka)K'_m(qS')}{\varepsilon_{r2} q K'_m(qa) I_m(ka) - \varepsilon_{r1} k K_m(qa) I'_m(ka)}\right], \quad (23)$$

where $\varepsilon_{r1} = \varepsilon_1/\varepsilon_0$ and $\varepsilon_{r2} = \varepsilon_2/\varepsilon_0$ are the dielectric constants, use has been made of the completeness relation (4). In the limit of infinity wavelengths, the behavior is dominated by the terms containing $I_m(ka)$ and $kI'_m(ka)$. Using the limiting property $I_m(x) \sim (x/2)^m/m!$, we find

$$f(\varphi) = e\left(\frac{\varepsilon_{r1}}{\varepsilon_{r2}} - 1\right) \sum_{m=1}^{\infty} \left[\frac{mK'_m(\kappa S')}{\varepsilon_{r2}\kappa a K'_m(\kappa a) - \varepsilon_{r1} K_m(\kappa a)}\right] \cos(m\varphi), \quad (24)$$

where the symmetry of (6) under the replacement $m \leftrightarrow -m$ has been used. Notice that, as expected, the net surface charge (obtained by integrating the above expression over φ) vanishes, since the rod is charge neutral. Note that, in the limit of a perfectly conductor cylinder, $\varepsilon_1 \to \infty$, Eq. (25) is simplified to

$$f(\varphi) = -\frac{e}{\varepsilon_2} \sum_{m=1}^{\infty} \left[\frac{mK'_m(\kappa S')}{K_m(\kappa a)}\right] \cos(m\varphi). \quad (25)$$

Fig. 2 shows the net induced charge per unit of polar angle, as predicted by Eq. (25), measured from a line joining the test particle and the rod's center. The rod has diameter $d = 20$ nm, and an internal dielectric constant of $\varepsilon_2 = 500$, whereas that of the aqueous outer environment is taken as $\varepsilon_1 = 80$. In 2a, the surface charge is shown at fixed ionic concentration $c_s = 1$ mM and several locations S where the test

particle is placed. In 2b, this quantity is shown for a test-particle at a fixed distance $S = 1.2a$ from the rod center, at different salt concentrations c_s. In all cases, an opposite charge is induced at the surface, with a sharp distribution around the line joining test particle and the rod center. The peak at $\varphi = 0$ become more pronounced as $S \to a$, indicating a short range attraction to the rod surface. Therefore, free ions close to the surface tend to be adsorbed due to its interaction with its own, oppositely charged images. Notice that the attraction becomes stronger as the ionic concentration decreases, as shown in Fig. 2. As a result, dissolved ions tend to be more adsorbed towards the tube surface when they average concentrations are lowered. This mechanism is due to the weaker screening of the wall-ion interaction by the intervening charges, leading to long range attraction of dissolved counterions into the surfaces.

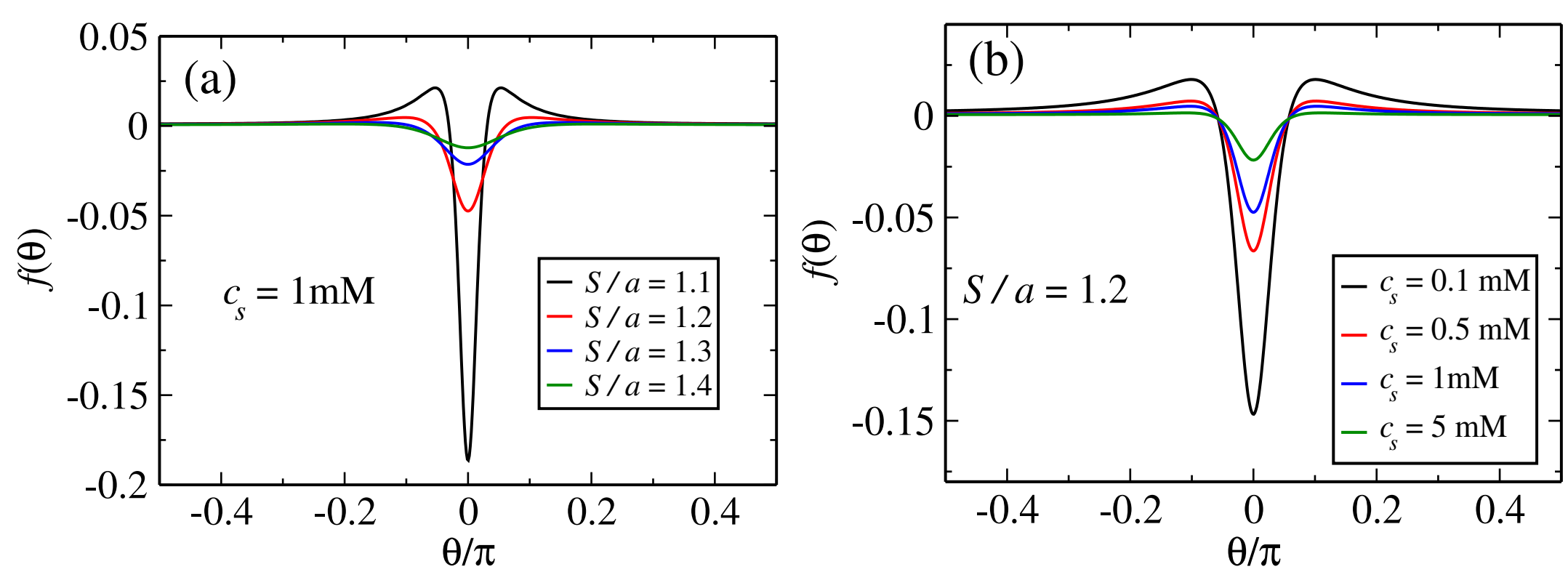

FIG. 2. Induced polarizing surface charge at a hard rod of dielectric constant $\varepsilon_2 = 500$, when a test-charge of magnitude q lies around its surface. In (a), the ionic concentration is fixed at $c_s = 1$ mM, while the test-charge distance S from the center of the tube is changed. In (b), the distance is fixed at $S = 1.2a$, and several concentrations of dissolved ions are considered.

It is clear from these considerations that a test-charge will be attracted towards

the rod surface, thereby inducing a static polarization charge at its surface. Such polarization of free electrons at the surface should clearly reduce the number of available free carriers for charge transport across the nanotubes. Moreover, the bulding-up of a layer of condensed cations onto the rod's surface should lead to an effective repulsion between them when the local environment becomes wet. In order to get a deeper insight into these mechanisms, let us now consider the interaction between the test-particle and the induced polarizing charge. This quantity can be readily evaluated as $eG(\mathbf{r} = \mathbf{r}', \mathbf{r}')$, *i. e.*, the point charge magnitude times the reaction potential evaluated at its own position $\boldsymbol{r}'$. Using Eq. (3), it is easy to check that the interaction potential $\Phi(S)$ between a test-charge e at S and the induced potential at the surface can be formally written as

$$\Phi(S) = \frac{4e^2}{\pi\varepsilon_2}\sum_{m=0}^{\infty}\left(1-\frac{\delta_{m0}}{2}\right)\int_0^{\infty}\frac{\varepsilon_2 q I_m(ka)I'_m(qa)-\varepsilon_1 k I_m(qa)I'_m(ka)}{\varepsilon_2 q K'_m(qa)I_m(ka)-\varepsilon_1 k K_m(qa)I'_m(ka)}\frac{K_m^2(qS)}{q^2S^2}dk. \tag{26}$$

Note that this potential is obviously isotropic: the angular dependence observed in Fig. 2 is averaged out, and ions will be attracted towards the surface regardless of their orientation with respect to the main axis. As a result, a system of mobile ions brought to the nearby vicinity of the surface should form a compact, isotropic layer of condensed charge around the rod's surface. In order to investigate this effect, we now proceed to calculate the *potential of mean force* $W_{im}(S)$, *i. e.*, the change in free energy resulting from adding an ion at a distance S from the rod center. This quantity can be easily evaluated by recognizing that the potential (26) is an averaged potential, which takes proper account of the thermal motion of surrounding charges that screens the reaction field. Therefore, the change in free-energy can be trivially

evaluated *via* a charging process, in which the charge of the test-particle is conceived as a coupling parameter that is meant to change from zero up to its real value. As a result of such a "charging process", we get:

$$W_{im}(S) = \frac{2e^2}{\pi\varepsilon_2}\sum_{m=0}^{\infty}\left(1-\frac{\delta_{m0}}{2}\right)\int_0^{\infty}\frac{\varepsilon_2 q I_m(ka)I'_m(qa)-\varepsilon_1 k I_m(qa)I'_m(ka)}{\varepsilon_2 q K'_m(qa)I_m(ka)-\varepsilon_1 k K_m(qa)I'_m(ka)}\frac{K_m^2(qS)}{q^2S^2}dk. \tag{27}$$

In the perfect screening limit $\varepsilon_1 \gg \varepsilon_2$, Eq. (39) can be further simplified to:

$$W_{im}(S) = \frac{2e^2}{\pi\varepsilon_2}\sum_{m=0}^{\infty}\left(1-\frac{\delta_{m0}}{2}\right)\int_0^{\infty}\frac{I_m(qa)K_m^2(kS)}{K_m(qa)q^2S^2}dk. \tag{28}$$

II. EQUILIBRIUM DISTRIBUTIONS AND POLARIZING CHARGES

As the degree of humidity surrounding the polymer/CNT composite grows larger, the hydrophilic nature of cellulose molecules leads to a local condensation of the incoming vapor, resulting in an increasingly wet environment around the network. Dissolved ions therein become free to flow alongside the solvent across the whole composite. The equilibrium transport of ions is thus dictated by a balance between their thermal motion and a local interaction with residual charges resulting from backbone dissociation, as well as the polarizing charges induced at the CNT surfaces, as outlined above. We expect the latter effect to be the dominant one in the present case, since the surface area available for ion contact from CNT is much larger than that of the polymer fibers. Therefore, we can focus on the local environment of a close vicinity to a CNT surface. The condition of vanishing net force that dictates

ionic thermal motion can be written as:

$$-k_B T \nabla \left[\log(\rho(S))\right] - e\nabla\psi(S) - \nabla W(S) = 0, \tag{29}$$

where k_B is the Boltzmann constant, T the local temperature, $\rho(S)$ and $\psi(S)$ are the averaged counterion density and averaged potential, respectively, both evaluated at a distance S from the rod's center. The first term above stands for the entropic force on the mobile counterions, free to move across the matrix due to local water diffusion. This force tries to prevent the building-up of strong ionic inhomogeneities, which clearly results in a large entropic cost. The remaining two terms, on the other hand, represent the electrostatic forces which tend to hold the mobile ions close to either suspended opposite charges or polarizing surfaces. It follows from Eq. (29) that the equilibrium distribution of dissolved counterions reads as

$$\rho(S) = c_s e^{-\beta(e\psi(S)+W(S))}, \tag{30}$$

where $\beta \equiv 1/k_B T$ is the inverse thermal energy, and c_s is the neutralizing background concentration (which clearly coincides with the bulk ion density at $S \to \infty$, due to overall charge neutrality). In this sense, the approach we adopt hereby is closely connected to the One Component Plasma (OCP) formalism, in which counterions are free to move over an inert background which neutralizes their net charge. The equilibrium distribution in (30) takes account of image charge contributions and ion solvation *via* the mean-force potential $W(S)$, which can be evaluated from Eq. (39). In order to evaluate the averaged potential $\psi(S)$ around a polarizing tube,

we employ a mean-field description in which the rod is placed at the center of a cylindrical cell of radius $R_c = a\sqrt{\phi}$, where a is the tube radius, and ϕ the averaged CNT packing fraction. Such condition ensures that the volume of the unit cell enclosing the rod is the same as the averaged volume occupied each individual rod in a lattice-like arrangement. The unit cell also contains mobile cations whose equilibrium distribution is given by Eq. (30). The ions move in rigid, neutralizing background of charge concentration $-c_s e$, which represents the effects from residual charges on the network. The whole cell is taken to be charge neutral. Combining the ionic distributions (30) with the Poisson equation inside the cell, the averaged potential can be computed via the Poisson-Boltzmann equation

$$\nabla^2\tilde{\psi} = \kappa^2 \left(e^{-(\tilde{\psi}(S)+\tilde{W}(S))} - 1\right), \tag{31}$$

where $\tilde{\psi} \equiv \beta e \psi$ and $\tilde{W} \equiv \beta W$ are dimensionless potentials. This equation can be numerically solved *via* a simple interactive algorithm, where boundary conditions of vanishing electric field and potential at the cell boundary, $\tilde{\psi}'(S = R_c) = 0$ and $\psi(S = R_c) = 0$. These conditions ensure the overall charge-neutrality inside the cell, whiling setting to zero the averaged potential due to external charges. Note that, in the absence of a reacting potential, $W(S) = 0$, Eq. (31) admits only the trivial solution $\tilde{\psi} = 0$ throughout the cell. The polarizing ion-surface interactions thus induces a non-uniform averaged potential around the rod, leading to an equilibrium cation distribution $\rho(S)$ which can be evaluated from (30) once Eq. (31) is numerically solved. Most importantly, an *averaged* polarization surface charge can be assigned to the CNT once the potential $\psi(S)$ on its vicinity is known. This quantity can be

formally written as $\sigma_{pol} = \boldsymbol{P} \cdot \boldsymbol{n}$, where $\boldsymbol{n} = \hat{\boldsymbol{e}}_S$ is the unit vector pointing outward the polarizing surface, and $\boldsymbol{P}$ is the averaged polarization. In terms of the mean potential $\psi(S)$, this quantity can be written as

$$\sigma_{pol} = -\frac{\varepsilon_2}{4\pi e}\frac{d\psi}{dS}\bigg|_{S=a_+}, \tag{32}$$

since the potential vanish inside the rod due to the rotation symmetry. The net effect is that the rod behaves as a charged surface which adsorbs surrounding cations. In the context of the conductivity properties of the polymer/CNT nanocomposite, such polarizing charges tend to reduce electron mobility inside the individual CNT, as well as to provide an effective repulsive interaction between neighboring tubes, increasing the inter-tube distance and thereby reducing network connectivity that provides a pathway for electronic transport through the matrix.

III. EFFECTIVE, COUNTERION-MEDIATED INTERACTIONS

Since the (undoped) nanotubes have no net charge, the layer of counterions condensed at their outer surface due to image-charge interactions has an overall effect of providing these objects with an effective net charge of decorating counterions. The situation is rather similar to a double-layer formation, where the polarizing charges at the inner surface play the role of a net negative charge that induce an outer layer of condensed counterions. The classical formalism of double layer interactions can thus be applied to investigate the counterion-induced pair interactions and structure of nanotubes embedded into a wet environment. This can be accomplished by considering the induced forces between the surface portions of two neighboring nanotubes

directly facing one another (as depicted in Fig. 1 of the main article). Since the nanotubes have a high aspect ratio, this setup can be, in a first-order approximation, devised as two planar charged surfaces of infinite size and same charges. Following the classical approach of double layer interactions, our strategy is to compute the repulse force between two such surfaces in the presence of intervening counterions and background charges, and then to apply Derjaguin's approximation to take account of curvature effects. To this end, we consider two flat plates of surface charge densities (per unit of elementary charge) σ_1 and σ_2, resulting from polarizing charges, located at positions $x = -D/2$ and $x = D/2$, respectively, where x is the perpendicular distance from the mid-plane separating the plates. It follows from the contact value theorem that the disjoint force per unit area Π between these surfaces can be computed as

$$\Pi = k_B T \sum_i [\rho_i(D/2) - \rho_i^b] + \frac{\varepsilon_2}{8\pi} \left(\frac{d\psi}{dx} \right)^2 \bigg|_{x=D/2} \approx k_B T \sum_i [\rho_i(x_0) - \rho_i^b], \qquad (33)$$

where ρ_i^b stands for the particle concentrations at the mid-plane when the surfaces are infinitely far apart, and x_0 is the position of vanishing electric field between the plates. While the first equality above is an *exact* statement, the second one is an approximation, which becomes exact in a mean-field description[b]. In the present case, $\rho_i^b = c_b$, and the disjoint pressure reads $\beta\Pi = \rho(x_0) - c_b$, where $\rho(x)$ is the cation concentration at position x. This quantity is most easily obtained in the context of a linear, Debye-Hückel (DH) approach, in which case the counterion distribution is approximated as $\rho(x) \approx c_b[1 - \beta e\psi(x)]$. The potential $\psi(x)$ in such a linear

[b] This approximation basically assumes that the sum in the second equality – which represents the net force per unit of area exerted on one surface – is constant throughout the inter-plane spacing, and thus can be evaluated at a point x_0 of vanishing electric field.

approximation satisfies the following DH equation

$$\frac{d^2\psi}{dx^2} = \kappa^2\psi. \tag{34}$$

This equation can be easily solved subject to the boundary conditions $\psi'(x = -D/2) = -\frac{4\pi e\sigma_1}{\varepsilon_2}$ and $\psi'(x = D/2) = \frac{4\pi e\sigma_2}{\varepsilon_2}$ at the charged plates. The solution reads

$$\psi(x) = \frac{4\pi e}{\varepsilon_2\kappa}\left[\frac{\sigma_1\cosh[\kappa(x-D/2)] + \sigma_2\cosh[\kappa(x+D/2)]}{\cosh(\kappa D)}\right]. \tag{35}$$

In order to evaluate the counterion concentration at x_0, it is convenient to multiply both sides of (34) by ψ' and integrate. This immediately yields $\kappa^2\psi^2 - (\psi')^2 = C$, where the integration constant C can be easily evaluated from Eq. (35) as

$$C = \left(\frac{4\pi e}{\varepsilon_2}\right)^2 \frac{[\sigma_1^2 + \sigma_2^2 + 2\sigma_1\sigma_2\cosh(\kappa D)]}{\cosh^2(\kappa D)}. \tag{36}$$

In particular, taking $x = x_0$ (the position at which ψ' vanishes), it follows that $\psi(x_0) = \pm\sqrt{C}/\kappa$. The corresponding ion concentration, in the linear approximation, reads $\rho(x_0) = c_b[1 - \beta e\psi(x_0)]$. Substitution into the last equality of Eq. (33) allows one to write the disjoint pressure $\beta\Pi$ (in the context of a linear approach) as

$$\beta\Pi = \frac{4\pi\lambda_B c_b}{\kappa\cosh(\kappa D)}\sqrt{(\sigma_1-\sigma_2)^2 + 4\sigma_1\sigma_2\cosh^2(\kappa D/2)}. \tag{37}$$

Let us now restrict our attention to the case where the approaching nanotubes are equally sized. In this case, the induced polarizing charges are the same, $\sigma_1 = \sigma_2 \equiv \sigma$,

and the above relation is significantly simplified as

$$\beta\Pi(D) = \frac{4\pi\lambda_B\sigma c_b}{\kappa\cosh(\kappa D/2)}. \tag{38}$$

Integration of this expression over the inter-plate distance D allows one to evaluate the pair interaction $W(D)$ per unit of area A between the flat surfaces in close contact as

$$\frac{\beta W(D)}{A} = \int_{-\infty}^{D} \beta\Pi(D')dD' = \frac{8\pi\lambda_B\sigma c_b}{\kappa^2}\ln\left(\frac{\sinh(\kappa D/2)}{1+\cosh(\kappa D/2)}\right) = 2\sigma\ln\left(\frac{1-e^{-\kappa D/2}}{1+e^{-\kappa D/2}}\right), \tag{39}$$

where the definition $\kappa = \sqrt{4\pi\lambda_B c_b}$ was used. Once the polarization surface charges σ for a given concentration of background charges c_b are known, the above expression provide us with an approximation for the double layer interactions between approaching nanotube surfaces. Now, according to Derjaguin's approximation, curvature effects on the force $F(D)$ between surfaces of two neighboring cylinders (of radii R_1 and R_2, oriented perpendicularly to each other) at a separation D can be incorporated *via* the relation

$$\beta F(D) = 2\pi\sqrt{R_1R_2}\frac{\beta W(D)}{A}. \tag{40}$$

It follows from this expression that a leading order curvature correction for the force $F(D)$ between two nanorods of same size diameter d, at a surface-to-surface distance

D, due to their double layer repulsion, can be written as

$$\beta F(D) = \frac{2\pi\sigma d}{\sin\theta} \ln\left(\frac{\sinh(\kappa D/2)}{1+\cosh(\kappa D/2)}\right), \tag{41}$$

where θ is the relative angle between the main axes of the nanotubes. This equation is equivalent to Eq. (7) of the main text for the dimensionless force $\tilde{F}$. The factor $1/\sin\theta$ is a first-order correction for the decreasing of the superimposed area when the rods have a relative orientation θ. A similar expression also holds for the short-ranged van-der-Waals attraction between these particles. At the same order of approximation, the double layer pair potential can be similarly computed. A direct substitution of Eq. (39) into Eq. (40), followed by integration over the inter-plate distances D, yields the following expression for the pair potential:

$$\beta u(D) = -\int \beta F(D')dD' = \frac{4\pi\sigma d}{\kappa \sin\theta}\left[\mathrm{Li}_2(-e^{-\kappa D/2}) + \mathrm{Li}_2(e^{-\kappa D/2})\right], \tag{42}$$

where $\mathrm{Li}_2(x)$ is the di-logarithm function. Again, the factor $1/\sin\theta$ compensates for the increased interaction when tubes have a relative orientation θ. This expression, obtained for the particular situation of equally sized rods, can be easily extended to incorporate size polydispersity, at least in situations where $\Delta\sigma = |\sigma_1 - \sigma_2|$ is not too large. As shown in the main text (see Fig. 5b), large deviations in such (polarizing) surface charges become prominent at higher salt concentrations, in which case the inverse screening length κ becomes large, and $\kappa D \gg 1$ for most of the relevant CNT distances. In either case, the first term in the square root in Eq. (37) can be neglected in comparison with the second one, such that the disjoint pressure in 38 becomes

unchanged under the replacement $\sigma \to \sqrt{\sigma_1\sigma_2}$. Moreover, it follows from Eq. (40) that the pair potential (42) within the same approximation can be extended to the interaction of polydisperse rods *via* the replacement $d \to \sqrt{d_1 d_2}$. As a result, Eq. (42) can, to a fairly accurate extent, be generalized as

$$\beta u_{ij}(D) \approx \frac{4\pi\sqrt{\sigma_i\sigma_j d_i d_j}}{\kappa \sin\theta}\left[\mathrm{Li}_2(-e^{-\kappa D/2}) + \mathrm{Li}_2(e^{-\kappa D/2})\right]. \tag{43}$$

Equations (42) and (43) need as input only the polarizing surface charges, which can be computed in the framework described above (see Eq. 32). Once this is accomplished, an estimation can be done for the averaged orientation θ between neighboring rods as follows. Consider two interacting rods which are allowed to freely rotate in response to their mutual influence. In the absence of external fields, we can take one of the rods with a fixed orientation (parallel to the z-axis in a spherical coordinate system), whereas the second one is free to rotate around the former. In this configuration, the probability of finding the rods at a relative orientation θ can be taken as

$$P_{ij}(\theta) = \frac{2\pi\int_0^\infty r^2 e^{-\beta u_{ij}(r,\theta)}}{\int e^{-\beta u_{ij}(r,\theta)} d\boldsymbol{r}} = \frac{\int_0^\infty r^2 e^{-\beta u_{ij}(r,\theta)} dr}{\int_{\theta_c}^{\pi/2} \sin\theta d\theta \int_0^\infty r^2 e^{-\beta u_{ij}(r,\theta)} dr}. \tag{44}$$

Here, the integration is performed over the center-of-mass coordinates from the two-particle system. Integration over polar angle θ is performed starting from a cut-off angle $\theta_c = \tan^{-1}(d/2L)$, which in practice does not represent a severe restriction, due to the large aspect ratio of rods under consideration. Eq. (44) is exact in the infinite dilution limit, where it represents the angular pair correlation function averaged over

all inter-particle distances. This expression is then used to analyze the averaged area $\langle A(\theta)\rangle = A_0\langle 1/\sin\theta\rangle$, shown in Fig. 7b in the main manuscript.

IV. AC IMPEDANCE SPECTROSCOPY AND APPARENT DIELECTRIC RESPONSE

A. Experimental AC conditions

Impedance spectra were acquired for three MWCNT/TOCNF films spanning the percolation region (Table I). Here, ϕ_v denotes the nominal MWCNT volume fraction on a dry-solid basis. Spectra were acquired from 20 mHz to 100 kHz, measured with a 20 mV rms perturbation. The near-threshold humidity series was acquired sequentially on the same device with unchanged contacts and electrical connections. These spectra do not constitute independent device replicates.

TABLE I. Films and humidity conditions included in the AC analysis.

Network regime	Nominal ϕ_v	RH (%)
Below threshold	0.183	49.8, 80.8
Near threshold	0.208	49.0, 81.0, 90.0, 94.5
Well percolated	0.341	49.5, 80.5

The films were measured in plane using two patterned metal contacts on a phenolic substrate; the coating covered both contacts and their intervening gap. WE and Sense were connected to one contact, and CE and RE to the other (Fig. 3). The sample and clips were inside the humidity chamber. The ground lead was connected to protective earth outside the chamber, not to the sample.

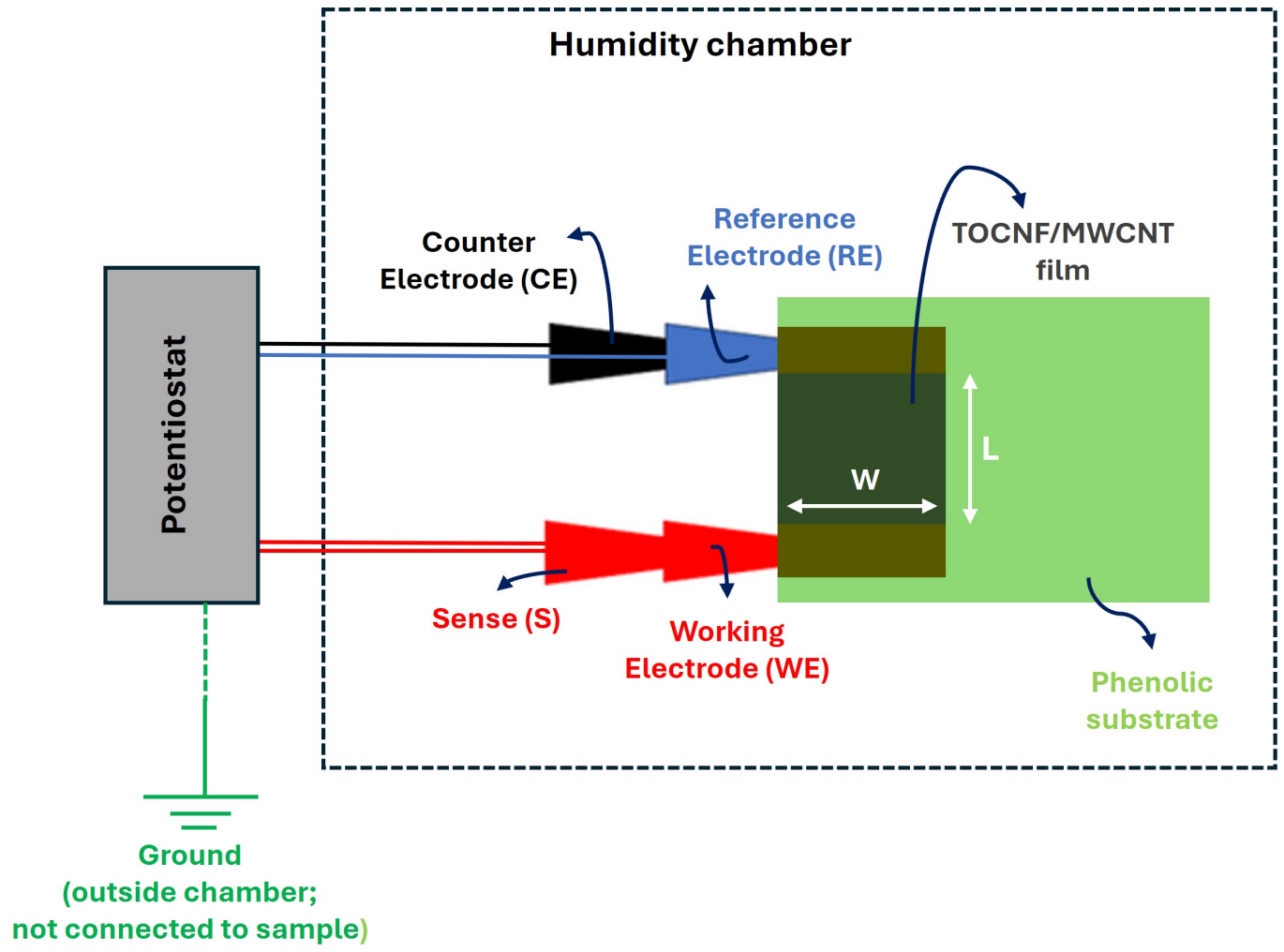

FIG. 3. Coplanar two-contact configuration. L is the gap between facing contact edges and W its transverse width. WE, Sense, CE, and RE identify the instrument leads; the ground connection is external to the chamber.

B. Calculation of the apparent electrical quantities

The measured impedance, $Z = Z' + iZ''$, was converted to admittance[1–4]:

$$Y = \frac{1}{Z} = G + iB, \qquad G = \frac{Z'}{Z'^2 + Z''^2}, \qquad B = \frac{-Z''}{Z'^2 + Z''^2}, \tag{45}$$

where G is conductance and B is susceptance. The plotted phase is $\theta = -\arg Z$; positive values denote capacitive sign.

The same nominal geometrical reference capacitance was used for all spectra:

$$C_{0,\text{nom}} = \varepsilon_0 \frac{t_{\text{nom}} W}{L}. \tag{46}$$

Here, ε_0 is the vacuum permittivity. The films were of micrometre-scale thickness,

but their thicknesses were not measured individually for the AC specimens. The below-threshold films were thinner than the percolated ones. We therefore used $t_{\mathrm{nom}} = 1\,\mu$m as a common reference for normalization, rather than assuming that every film had this thickness. With $L = W = 1.0$ cm, this gives $C_{0,\mathrm{nom}} \simeq 8.854\times10^{-18}$ F. This normalization preserves the measured frequency and humidity trends, but does not correct for thickness differences when comparing apparent conductivity and permittivity across compositions.

Using $\omega = 2\pi f$ and $\varepsilon^*_{\mathrm{app}} = \varepsilon'_{\mathrm{app}} - i\varepsilon''_{\mathrm{app}}$:

$$\sigma'_{\mathrm{app}} = \frac{\varepsilon_0}{C_{0,\mathrm{nom}}}G, \qquad \varepsilon'_{\mathrm{app}} = \frac{B}{\omega C_{0,\mathrm{nom}}}, \tag{47}$$

$$\varepsilon''_{\mathrm{app}} = \frac{G}{\omega C_{0,\mathrm{nom}}}, \qquad \tan\delta_{\mathrm{app}} = \frac{G}{B} \quad (B \neq 0). \tag{48}$$

The coplanar field samples the film, substrate, and surrounding medium. Without electrostatic calibration and open/blank measurements, the contributions of the film, contacts, and apparatus cannot be separated. These quantities are therefore reported as apparent, not intrinsic material properties. Phase and G/B do not depend on the nominal geometry, but still describe the complete measured configuration.

C. Results and interpretation

The main-text figure includes all 21 measured points between 20 mHz and 2 Hz. For the numerical comparison between humidity conditions, we used the median conductance of the 11 points between 20 and 200 mHz.

Humidification increases conductance approximately 8.2-fold below percolation,

but decreases it by approximately 32% and 25% near and well above the threshold, respectively. Together with the component controls in the main text and the reported humidity-dependent ionic conduction of TOCNF[6], this reversal supports a change from matrix-assisted ionic transport to CNT-network-dominated electronic transport. These relative changes were calculated directly from conductance, without using the nominal thickness. The comparison does not demonstrate humidity-induced loss of percolation within a single device.

The low-frequency phase response is shown in Fig.4b of the main text. For each spectrum, the median phase was calculated over the 11 measured frequencies between 20 and 200 mHz. For the near-threshold film, this median reaches 0.21° at 90% RH and 1.02° at 94.5% RH, whereas the well-percolated controls remain close to zero. This humidity-dependent response is consistent with charge redistribution, but does not establish whether polarization occurs in the matrix, at CNT interfaces, or at the electrodes.

The full spectra of the percolating films are presented in Fig. 4. Conductivity is approximately constant at low frequency, with larger deviations at higher frequency, particularly at high RH. Because $\varepsilon''_{\mathrm{app}} = \sigma'_{\mathrm{app}}/(\varepsilon_0\omega)$, a nearly constant conductivity gives $\varepsilon''_{\mathrm{app}} \propto 1/\omega$. The large low-frequency loss thus contains a conductive contribution and is not, on its own, evidence of interfacial polarization.

When phase is close to zero, the small B component can change sign. Since $G > 0$ in these six spectra, negative $\varepsilon'_{\mathrm{app}}$ and $\tan\delta_{\mathrm{app}}$ correspond to $B < 0$ ($Z'' > 0$), not negative dissipation. They are not assigned to intrinsic negative permittivity. Likewise, large fluctuations in G/B can arise from division by a small B, without indicating a resolved relaxation.

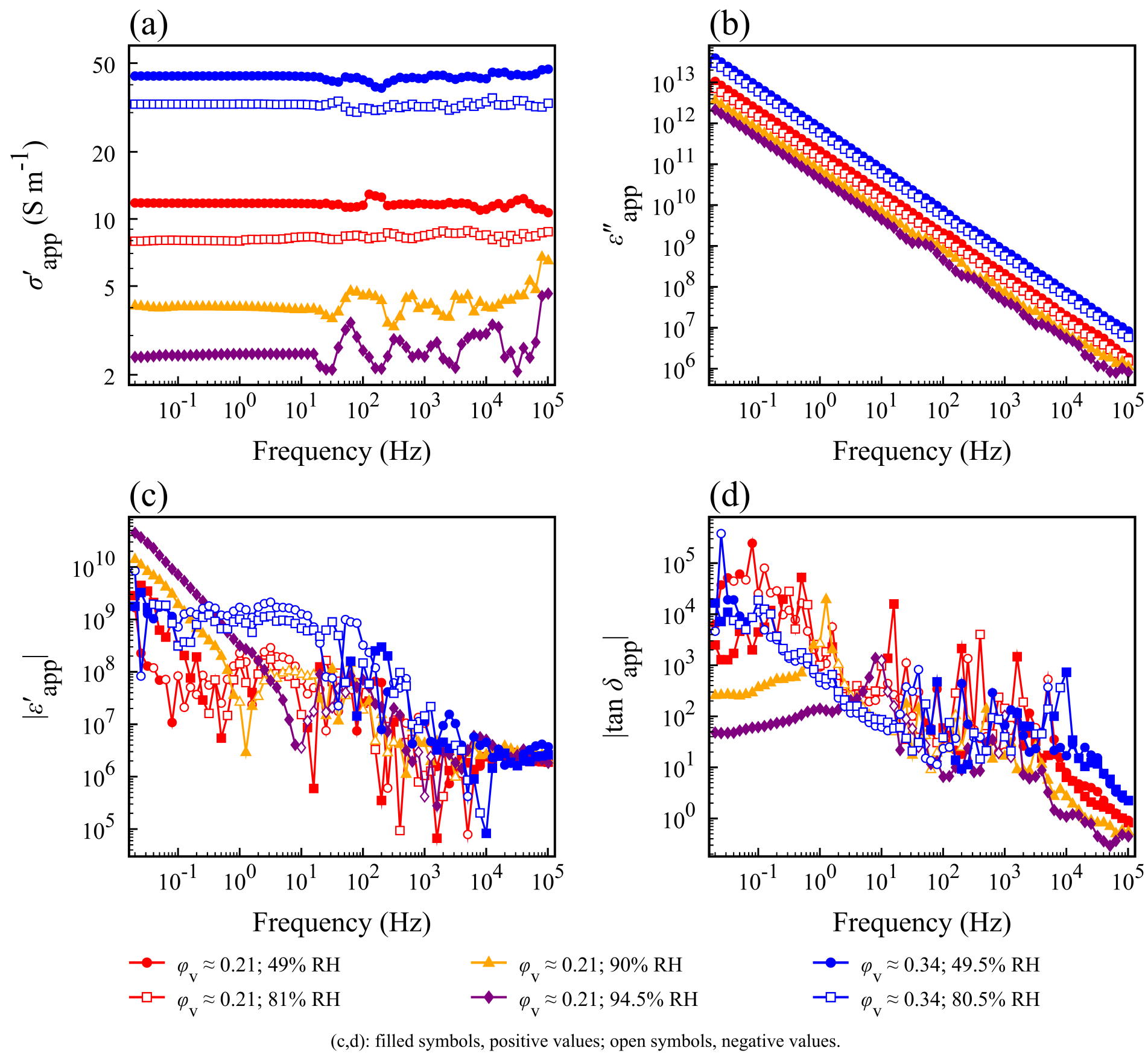

FIG. 4. Full spectra of the near-threshold and well-percolated films: (a) apparent conductivity, (b) apparent dielectric loss, (c) magnitude of apparent real permittivity, and (d) magnitude of apparent loss tangent. In (c) and (d), filled and open symbols denote positive and negative algebraic values, respectively; magnitudes permit logarithmic plotting. Lines join measured points without smoothing.

The below-threshold records (Fig. 5) contain seven negative-Z' points at 49.8% RH and three at 80.8% RH. These points are retained but not interpreted as passive material behavior. Both spectra have positive Z' throughout 20 mHz–2 Hz, although their phase is substantially scattered. Their conductance trend is used for

comparison, not to extract intrinsic dielectric properties or a relaxation time.

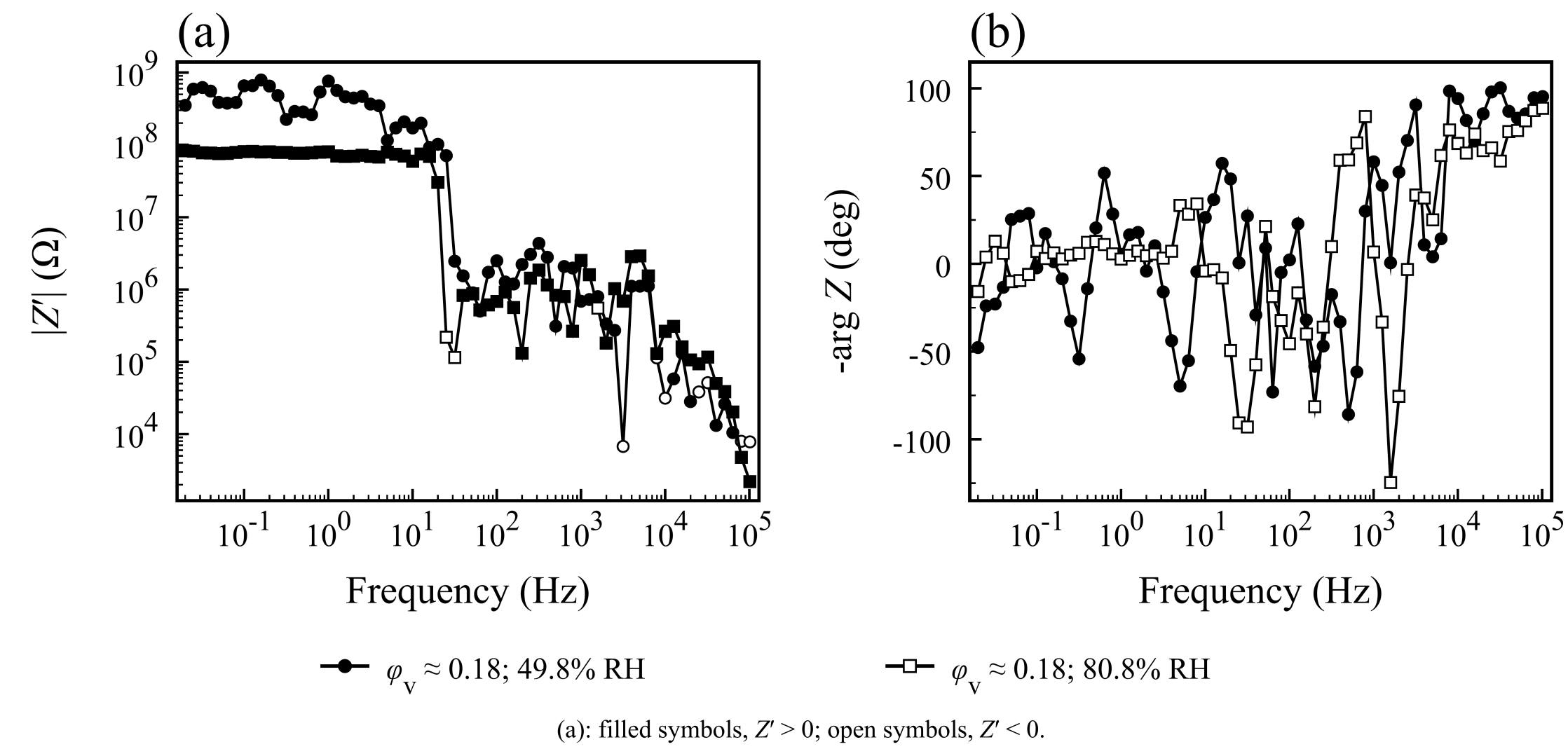

FIG. 5. Full below-threshold spectra at 49.8 and 80.8% RH: (a) magnitude of real impedance and (b) signed phase. In (a), filled and open symbols denote positive and negative Z', respectively. The main-text comparison uses 20 mHz–2 Hz, where $Z' > 0$ in both conditions.

The AC results help to distinguish the transport regimes, but do not independently confirm the microscopic counterion distribution predicted by the equilibrium model.

V. ELECTRIC FORCE MICROSCOPY MEASUREMENTS

Electric Force Microscopy measurements Electrical force microscopy (EFM) is a two-pass scanning probe technique in which the surface topography is first acquired by conventional AFM (intermittent contact mode) and then, in a second pass, the conductive tip subsequently is retracted at a constant lift height and scan the same line. A DC bias (V_{EFM}) is applied between the tip and the sample, and the resulting electrostatic force gradient is detected through the shift in the cantilever resonance frequency ($\Delta\omega$). Thus, the EFM response reflects the electrostatic interaction between the tip and the sample, including contributions associated with local capacitance, surface potential, and charge.

In our measurements, a lift height of 50 nm was used, substantially reducing short-range tip–sample interactions and making the measured signal primarily sensitive to long-range electrostatic interactions. In the present system, the electrostatic interaction involves the SiO_2 substrate, the TEMPO-oxidized cellulose nanofibers (TOCNF), and the multiwall carbon nanotubes (MWCNTs). Therefore, the measured EFM contrast contains both capacitive and electrostatic contributions. The capacitive contribution is primarily determined by the local geometry and dielectric properties of the system and, in the absence of a polarity-dependent electrostatic potential, is expected to be approximately symmetric upon reversal of the applied bias. In contrast, a non-zero local electrostatic potential associated with charge redistribution can introduce a polarity-dependent contribution to the measured response.

To experimentally investigate this effect, we acquired EFM images of the same region using opposite DC tip biases of +8 V and −8 V, while maintaining an identical

lift height of 50 nm. This technique is able to accurately resolve the tip swamping region into spatial dimensions where MWCNT are clearly identified, as the one highlighted in Fig 6(a).

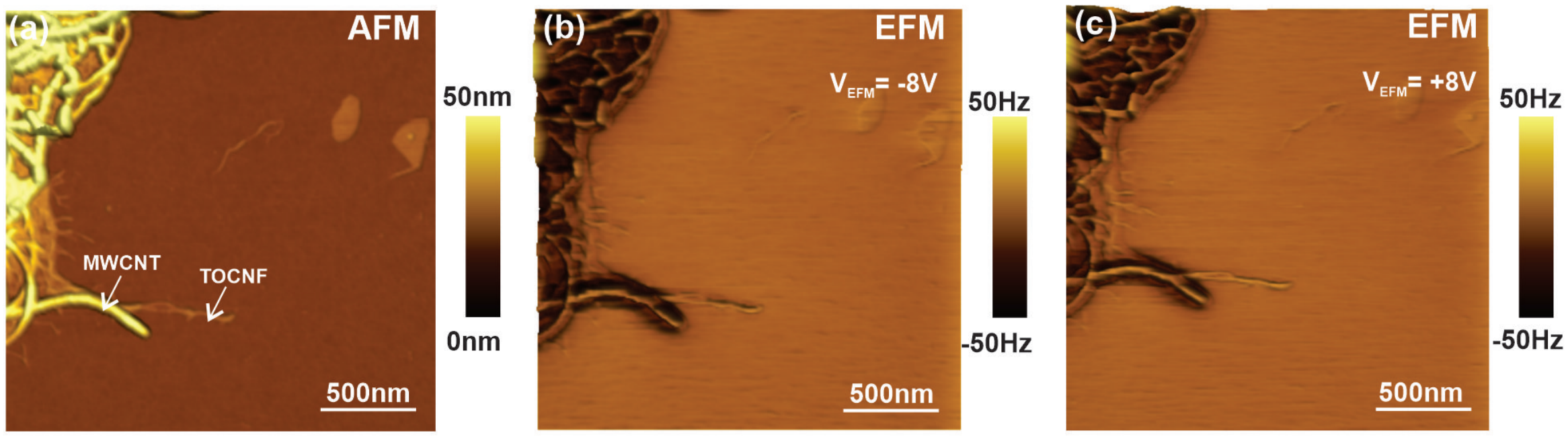

FIG. 6. AFM and EFM characterization of the MWCNT/TOCNF nanocomposite (S3 ink). (a) AFM topography showing a representative MWCNT and a TOCNF deposited on a SiO_2 substrate. (b,c) EFM frequency-shift images acquired over the same region with DC tip biases of −8 V and +8 V, respectively, using lift height=50 nm. The EFM frequency shift is proportional to the electrostatic force gradient and reflects the combined influence of local capacitance and surface potential.

The newly added Figure 6 also shows that the EFM contrast associated with the MWCNT regions varies upon reversal of the applied bias. Quantitative comparison shows that the frequency shift measured with a positively biased tip is approximately 20% lower than that obtained with a negatively biased tip under otherwise identical experimental conditions. Because the measurements were performed over the same region and at the same lift height, this difference cannot be attributed simply to variations in sample morphology between the two measurements. The observed polarity dependence indicates that the electrostatic response contains a contribution from a non-zero local electrostatic potential in the MWCNT/TOCNF region, in addition to the polarity-independent capacitive contribution. Reversal of the tip

polarity changes the electrostatic interaction with the interfacial charge distribution and consequently modifies the measured electrostatic force gradient. According to our proposed mechanism, the negatively charged carboxylate groups of the TOCNF retain Na^+ counterions in the vicinity of the conductive MWCNT surface. Their accumulation at the CNT/TOCNF interface can generate a localized interfacial electrostatic potential and promote charge redistribution within the nanotube. The resulting electrostatic environment is therefore expected to respond differently to opposite tip biases, as observed experimentally. Importantly, however, EFM cannot directly determine the chemical identity or spatial distribution of the ions responsible for this electrostatic response. We therefore interpret the polarity-dependent EFM response as experimental evidence for a localized interfacial electrostatic potential that is consistent with the proposed Na^+ polarized interfacial structure. In combination with the known presence of Na counterions associated with the TEMPO-oxidized cellulose and the electrical behavior observed for the MWCNT/TOCNF composites, these results support the proposed counterion-mediated polarization mechanism and the formation of an EDL-like interfacial structure[5].

VI. DEVICE-TO-DEVICE REPRODUCIBILITY AND SUB-PERCOLATION CONTROLS

A. Scope of the representative I–V measurements

The I–V curves in Figure 3 of the main manuscript were acquired to establish the electrical behavior of representative devices and are not used as device-to-device statistics. In particular, the two sub-percolation curves in Figure 3d are qualita-

tive controls from one device per composition. The nominal volume fractions were calculated from nominal dry component masses and densities and should not be interpreted as the in situ volume fraction of a porous, hydrated coating.

TABLE II. Composition and scope of the sub-percolation controls in Figure 3d.

Curve	MWCNT:TOCNF	MWCNT (dry wt%)	Nominal ϕ_v	n	Use
Blue	0.15:1	12.8	0.1152	1	Qualitative control
Green	0.21:1	17.2	0.1555	1	Qualitative control

B. Statistical treatment of the steady-state humidity response

Steady-state reproducibility was evaluated using three independently coated devices for each formulation (S1, S2, and S3). For each device, the resistance gain was calculated relative to its own low-humidity plateau as $R_G = 100(R - R_0)/R_0$. Only RH plateaus represented in all three devices were retained. The values plotted in revised Figure 4a are the arithmetic mean and sample standard deviation across the three devices; The error bars describe the spread of the complete devices, including coating and contact variability; they are not repeated readings of one device.

TABLE III: Individual steady-state values used to calculate the statistics plotted in revised Figure 4a. Each device column reports RH/R_G (both in %). The last two columns report the mean ± sample standard deviation across the available devices. A dash denotes that the corresponding device was not measured at that plateau.

	Individual RH/R_G values (%/%)				Across devices	
Plateau	Device 1	Device 2	Device 3	n	Mean RH ± SD (%)	Mean R_G± SD (%)
**S1 (MWCNT:TOCNF = 1:0.5)**						
1	19.7/0.0	19.8/0.0	17.8/0.0	3	19.1 ± 1.1	0.0 ± 0.0
2	30.4/0.9	29.9/−1.0	28.8/1.3	3	29.7 ± 0.8	0.4 ± 1.2
3	40.3/2.1	39.9/2.5	41.4/3.8	3	40.5 ± 0.8	2.8 ± 0.9
4	50.4/4.2	50.4/1.9	47.9/5.4	3	49.6 ± 1.4	3.8 ± 1.8
5	60.0/6.5	59.9/3.0	59.6/9.1	3	59.8 ± 0.2	6.2 ± 3.1
6	69.9/10.4	70.0/9.7	72.0/15.0	3	70.6 ± 1.2	11.7 ± 2.9
7	80.3/19.2	80.0/23.5	79.0/20.7	3	79.8 ± 0.7	21.1 ± 2.2
8	85.2/26.1	85.4/34.7	–	2	85.3 ± 0.1	30.4 ± 6.1
9	90.5/47.7	90.4/65.9	90.4/44.0	3	90.4 ± 0.1	52.5 ± 11.7
10	94.7/81.7	95.6/76.3	95.5/76.3	3	95.3 ± 0.5	78.1 ± 3.1
**S2 (MWCNT:TOCNF = 1:1.7)**						

	Individual RH/R_G values (%/%)				Across devices	
Plateau	Device 1	Device 2	Device 3	n	Mean RH ± SD (%)	Mean R_G± SD (%)
1	17.3/0.0	19.2/0.0	19.0/0.0	3	18.5 ± 1.0	0.0 ± 0.0
2	27.5/1.0	30.3/1.8	30.3/2.3	3	29.4 ± 1.6	1.7 ± 0.6
3	38.6/3.6	40.3/4.7	39.3/5.4	3	39.4 ± 0.9	4.6 ± 0.9
4	48.8/6.8	50.2/8.9	49.8/9.6	3	49.6 ± 0.7	8.4 ± 1.5
5	59.7/11.1	60.1/14.4	59.3/15.2	3	59.7 ± 0.4	13.6 ± 2.2
6	70.0/17.3	70.2/25.6	69.9/25.1	3	70.0 ± 0.2	22.6 ± 4.6
7	80.2/29.8	80.0/43.6	79.9/43.8	3	80.0 ± 0.2	39.1 ± 8.0
8	89.4/62.5	89.7/88.0	89.9/96.9	3	89.7 ± 0.3	82.5 ± 17.8
9	95.3/181.5	94.6/166.2	95.2/208.7	3	95.0 ± 0.4	185.5 ± 21.5
**S3 (MWCNT:TOCNF = 1:2.2)**						
1	18.3/0.0	18.8/0.0	18.4/0.0	3	18.5 ± 0.3	0.0 ± 0.0
2	28.2/1.9	29.9/1.9	28.0/1.5	3	28.7 ± 1.0	1.8 ± 0.2
3	39.9/6.2	39.6/5.2	39.0/5.4	3	39.5 ± 0.5	5.6 ± 0.5
4	48.9/10.3	50.0/10.1	48.9/9.6	3	49.3 ± 0.6	10.0 ± 0.3
5	61.4/15.7	59.5/15.8	59.9/16.3	3	60.3 ± 1.0	15.9 ± 0.3
6	71.8/21.2	70.3/27.0	70.0/24.1	3	70.7 ± 1.0	24.1 ± 2.9
7	82.3/52.5	79.9/49.2	78.6/42.9	3	80.3 ± 1.9	48.2 ± 4.8

	Individual RH/R_G values (%/%)				Across devices	
Plateau	Device 1	Device 2	Device 3	n	Mean RH ± SD (%)	Mean R_G± SD (%)
8	–	90.1/124.1	90.4/120.9	2	90.2 ± 0.2	122.5 ± 2.3
9	94.8/270.9	95.1/251.3	95.0/244.5	3	95.0 ± 0.2	255.6 ± 13.7

At the highest RH plateau measured in all three devices, the resistance gains were $78.1 \pm 3.1\%$ for S1, $185.5 \pm 21.5\%$ for S2, and $255.6 \pm 13.7\%$ for S3 (mean ± sample standard deviation, $n = 3$).

TABLE IV. Device and contact configurations included in the steady-state $n = 3$ analysis.

Ink	MWCNT:TOCNF	Device	Contact
S1	1:0.5	1	Cu
S1	1:0.5	2	Cu
S1	1:0.5	3	Au-protected Cu
S2	1:1.7	1	Cu
S2	1:1.7	2	Au-protected Cu
S2	1:1.7	3	Au-protected Cu
S3	1:2.2	1	Cu
S3	1:2.2	2	Au-protected Cu
S3	1:2.2	3	Au-protected Cu

VII. STEP RESPONSE, RECOVERY, AND WITHIN-SESSION CYCLING

A. Step-response protocol and 10–90% calculation

Dynamic measurements were performed under a fixed 0.2 V bias while the chamber was switched between low- and high-humidity gas-flow conditions. Chamber RH and device resistance were recorded simultaneously. The analysis includes one S2 device and one S3 device. Every transition in Table V comes from a record maintained at 0.2 V throughout.

For each complete transition, the initial resistance was the median of the first 2 s of the selected segment. The final resistance was the median of a 5 s window ending 5 s before the segment ended, so that the final value represented the new plateau rather than the switching boundary. The 10% and 90% levels were calculated from these two plateaus. The apparent response or recovery time was the interval between the first crossings of those levels, $t_{10-90} = t_{90} - t_{10}$. The same procedure was applied to the simultaneously recorded chamber RH. No kinetic fit was used, and only transitions containing both stable plateaus were accepted.

Because humidity inside the chamber changed on a similar timescale, the measured values describe the response of the device–chamber system rather than an intrinsic time constant of the film. The present data are not sufficient for a quantitative comparison of the intrinsic response rates of S2 and S3.

For comparison, Zhu et. al.[7] reported response and recovery times of 322 and 442 s for a vacuum-filtered TEMPO-oxidized cellulose-nanofiber/CNT film over an 11–95% RH excursion. Although the protocols differ, both studies show responses

on the scale of minutes. This comparison is only qualitative.

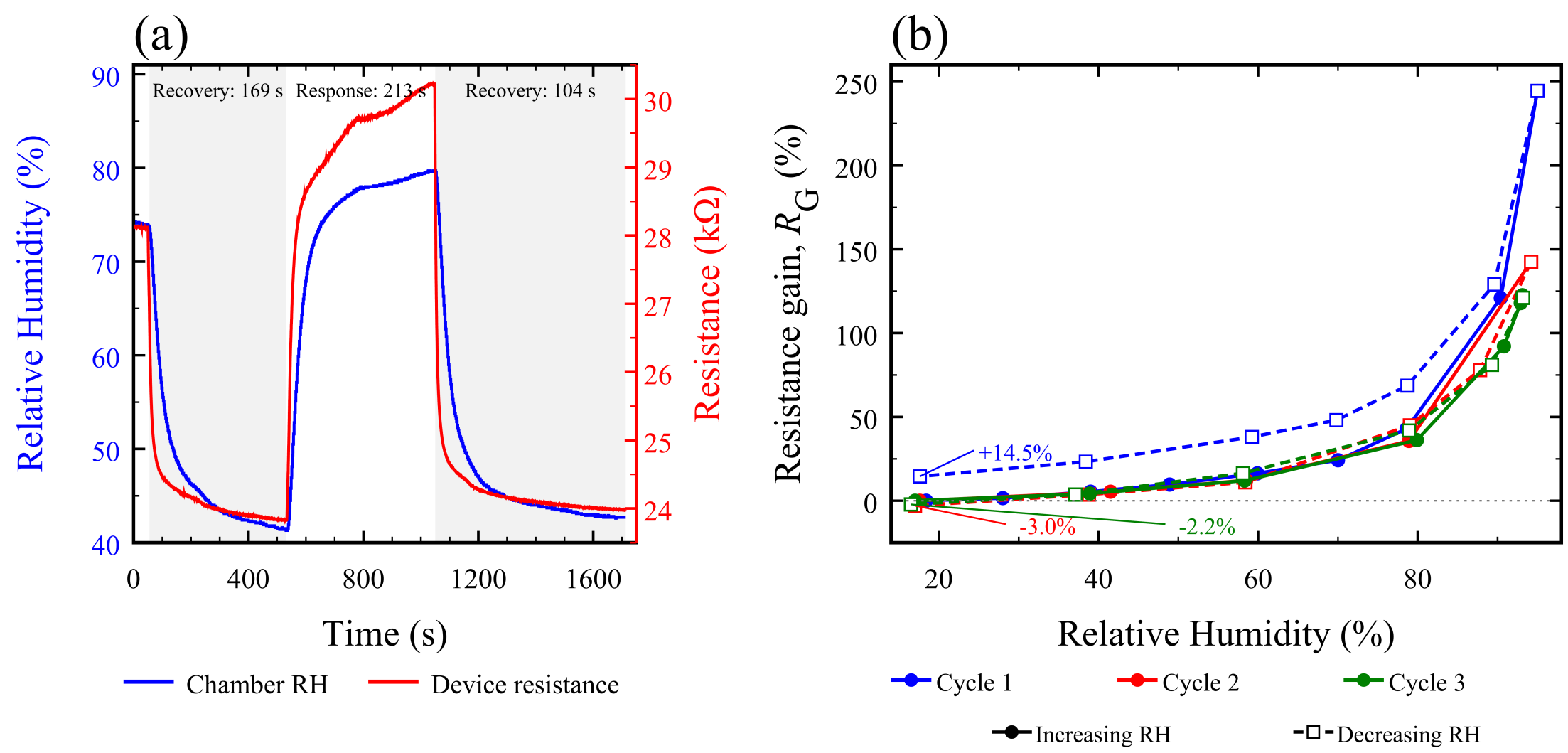

FIG. 7. Dynamic humidity response and within-session cycling. (a) Representative uninterrupted measurement on one S3 device at 0.2 V; chamber RH (left axis) and resistance (right axis) were recorded simultaneously. Shaded regions identify the analyzed transitions, and the labels indicate the time required for the resistance to change from 10% to 90% of its total variation. (b) Three consecutive increasing/decreasing-RH cycles on one S3 device. Each cycle is normalized to its low-RH resistance at the start of that cycle; endpoint labels give the residual baseline change. Lines are guides to the eye.

TABLE V: Individual 10–90% response and recovery times. Results for S2 and S3 are reported separately because they correspond to different formulations.

Ink	Device	Segment	Direction	Device (s)	Chamber (s)
S2	C	Run 1 / rise	RH increase	121.8	138.9
S2	C	Run 1 / fall	RH decrease	158.1	142.8
S2	C	Run 2 / fall 1	RH decrease	89.8	148.9

Ink	Device	Segment	Direction	Device (s)	Chamber (s)
S2	C	Run 2 / rise 1	RH increase	183.8	155.0
S2	C	Run 2 / fall 2	RH decrease	86.1	130.3
S2	C	Run 2 / rise 2	RH increase	114.0	122.3
S3	B	Run 1 / fall	RH decrease	40.7	114.7
S3	B	Run 2 / fall 1	RH decrease	168.9	189.3
S3	B	Run 2 / rise 1	RH increase	212.6	148.9
S3	B	Run 2 / fall 2	RH decrease	104.3	148.9

B. Three-cycle increasing/decreasing-RH experiment

One S3 device was subjected to three consecutive increasing/decreasing-RH cycles in a single experimental session. For every point in a given cycle, including both the increasing- and decreasing-RH branches, $R_G = (R - R_0)/R_0 \times 100\%$, where R_0 is the low-RH resistance measured at the beginning of that cycle. After the first cycle, the resistance measured upon returning to low RH remained 14.5% above its initial value. The corresponding changes after cycles 2 and 3 were -3.0% and -2.2%, respectively. The largest separation between matched increasing- and decreasing-RH points decreased from 25.7 percentage points in the first cycle to 9.3 and 5.7 percentage points in cycles 2 and 3. Relative to the full response of each cycle, these values correspond to 10.5%, 6.5%, and 4.7%, respectively.

After the initial conditioning cycle, the device showed consistent response profiles and recovered its low-RH baseline to within 3.0% and 2.2% during the subsequent

cycles. This behavior was maintained over several hours of continuous measurement, demonstrating short-term operational stability and repeatability within the experimental session. In addition, the liquid inks remained visually stable and homogeneous, without visible phase separation, after more than 12 months of refrigerated storage. This observation provides qualitative evidence of the long-term storage stability of the dispersions, which is relevant to their practical handling and processing. The cycling experiment and the storage observation address different aspects of stability: the former concerns the electrical response of the device, whereas the latter concerns the colloidal stability of the ink before deposition.

TABLE VI. Cycle-specific response range, residual low-RH offset, and largest matched-branch separation.

Cycle	Maximum R_G (%)	Final offset (%)	Maximum separation (p.p.)	Relative separation (%)
1	244.5	+14.5	25.7	10.5
2	142.5	−3.0	9.3	6.5
3	122.6	−2.2	5.7	4.7

REFERENCES

[1]Lazanas, A. Ch.; Prodromidis, M. I. Electrochemical Impedance Spectroscopy—A Tutorial. *ACS Meas. Sci. Au* **2023**, *3*, 162–193. `https://doi.org/10.1021/acsmeasuresciau.2c00070`.

[2]Lazanas, A. Ch.; Prodromidis, M. I. Correction to "Electrochemical Impedance Spectroscopy—A Tutorial". *ACS Meas. Sci. Au* **2025**, *5*, 156. `https://doi.org/10.1021/acsmeasuresciau.5c00007`.

[3]Philipose, U.; Jiang, Y.; Western, B.; Harcrow, M.; Littler, C.; Sood, A.; Zeller,

J. W.; Lineberry, B.; Syllaios, A. J. Impedance Analysis and Noise Measurements on Multi Walled Carbon Nanotube Networks. *Materials* **2021**, *14*, 7509. https://doi.org/10.3390/ma14247509.

[4]Edwards, D. D.; Hwang, J.-H.; Ford, S. J.; Mason, T. O. Experimental Limitations in Impedance Spectroscopy: Part V. Apparatus Contributions and Corrections. *Solid State Ionics* **1997**, *99*, 85–93. https://doi.org/10.1016/S0167-2738(97)00206-3.

[5]Barboza, A. P. M.; Gomes, A. P.; Chacham, H.; Neves, B. R. A. Probing Electric Characteristics and Sorting Out Metallic from Semiconducting Carbon Nanotubes. *Carbon* **2010**, *48*, 3287–3292. https://doi.org/10.1016/j.carbon.2010.05.028.

[6]Zhu, J.; Zhu, P.; Zhu, Y.; Ye, Y.; Sun, X.; Zhang, Y.; Rojas, O. J.; Servati, P.; Jiang, F. Surface Charge Manipulation for Improved Humidity Sensing of TEMPO-Oxidized Cellulose Nanofibrils. *Carbohydrate Polymers* **2024**, *335*, 122059. https://doi.org/10.1016/j.carbpol.2024.122059.

[7]Zhu, P.; Liu, Y.; Fang, Z.; Kuang, Y.; Zhang, Y.; Peng, C.; Chen, G. Flexible and Highly Sensitive Humidity Sensor Based on Cellulose Nanofibers and Carbon Nanotube Composite Film. *Langmuir* **2019**, *35*, 4834–4842. https://doi.org/10.1021/acs.langmuir.8b04259.
